# The search for an optimal tissue‐engineered urethra model for clinical application based on preclinical trials in male animals: A systematic review and meta‐analysis

**DOI:** 10.1002/btm2.10700

**Published:** 2024-07-23

**Authors:** Natalia Chepelova, Guzel Sagitova, Daniel Munblit, Aleksandr Suvorov, Andrey Morozov, Anastasia Shpichka, Peter Glybochko, Peter Timashev, Denis Butnaru

**Affiliations:** ^1^ Institute for Regenerative Medicine, Sechenov First Moscow State Medical University (Sechenov University) Moscow Russia; ^2^ Care for Long Term Conditions Division, Florence Nightingale Faculty of Nursing, Midwifery and Palliative Care King's College London London UK; ^3^ Department of Paediatrics and Paediatric Infectious Diseases, Institute of Child's Health, Sechenov First Moscow State Medical University (Sechenov University) Moscow Russia; ^4^ Office of Scientific Development and Clinical Research, Sechenov First Moscow State Medical University (Sechenov University) Moscow Russia; ^5^ Institute for Urology and Reproductive Health, Sechenov First Moscow State Medical University (Sechenov University) Moscow Russia

**Keywords:** laboratory animal models, reconstructive urethroplasty, regenerative medicine, tissue engineering, urethral stricture

## Abstract

Tissue engineering has emerged as a promising avenue for reconstructive urology, though only a limited number of tissue‐engineered urethral constructs have advanced to clinical testing. Presently, there exists a dearth of agreement regarding the most promising constructs deserving of implementation in clinical practice. The objective of this review was to provide a comprehensive analysis of preclinical trials findings of a tissue‐engineered urethra and to identify the most promising constructs for future translation into clinical practice. A systematic search of the Pubmed, Scopus, and PMC databases was conducted in accordance with the PRISMA statement. Manuscripts published in English between 2015 and 2022, reporting on the methodology for creating a tissue‐engineered urethra, assessing the regenerative potential of the scaffold in a male animal model, and evaluating the clinical and histological outcomes of treatment, were included. A total of 48 manuscripts met the inclusion criteria, with 12 being eligible for meta‐analysis. Meta‐analysis revealed no significant benefit of any matrix type in terms of complication rates. However, acellular matrices demonstrated significant advantage over cellular matrices in case of no postoperative stricture formation (odds ratio = 0.06 [95% CI 0.01; 0.23], *p* < 0.01). Among all subgroups (animal models and scaffold types), the usage of acellular matrices resulted in advantageous effects. The meta‐regression analysis did not show a significant impact of defect length (β1 = −0.02 [−0.28; 0.23], *p* = 0.86). We found that decellularized materials may carry less relevance for urethral reconstruction due to unfavorable preclinical outcomes. Natural polymers, used independently or with synthetic materials, resulted in better postoperative outcomes in animals compared to purely synthetic constructs. Acellular scaffolds showed promising outcomes, matching or exceeding cellular constructs. However, more studies are needed to confirm their clinical effectiveness.

**Abbreviations**
ACA: acellular carotid artery
ADM: acellular dermal matrix
ADSC: adipose‐derived stromal cells
ADSC‐exos: adipose‐derived stromal cells' exosomes
AM: amniotic membrane
AMSC: amniotic mesenchymal cells
BC: bacterial cellulose
BF: buccal flap
BMG: buccal mucosa graft
BMSC: bone marrow stromal cells
BrdU: bromodeoxyuridine
BSM: bladder submucosa matrix
BSMh: bladder submucosa matrix hydrogel
CBD‐VEGF: collagen‐binding VEGF
CCC: collagen cell carriers
CH: collagen hydrogel
CPO: calcium peroxide
cPUU: polyurethane‐urea coated by collagen
dCGT: double‐layered collagen gel tubes
DMBC/SPI: double‐modified bacterial cellulose/soybean protein isolate
EPC: endothelial progenitor cells
FB: fibroblasts
FGFR2: fibroblast growth factor receptor 2
GT: granulation tissue
HA: hyaluronic acid
HACC: 2‐hydroxypropyltrimethyl ammonium chloride chitosan
HD graft: high fiber density collagen graft
LD graft: low fiber density collagen graft
LSE: living skin equivalent
MC: mesothelial cells
MSC: mesenchymal stromal cells
NSC: nonsurgical control
OK: oral keratinocytes
PAA: peracetic acid
PCL: poly(ε‐caprolactone)
PEG: poly(ethylene glycol)
PLA: polylactid acid
PLC: poly‐l‐lactide‐caprolactone
PLG: poly‐l‐lactide‐glycolide
PL‐PC: poly‐d,l‐lactide/poly‐ε‐caprolactone
PTMC: poly(trimethylene carbonate)
PU‐alt: alternating block polyurethane
PU‐run: polyurethane block copolymer
PUU: polyurethane‐urea
SDF‐1α: stromal cell‐derived factor‐1 alpha
SF: silk fibroin
SIS: small intestinal submucosa
SMC: smooth muscle cells
UC: urothelial cells
USC: urine‐derived stem cells
USPIO: ultrasmall super‐paramagnetic iron oxide

**Translational Impact Statement**

Within the domain of urological tissue engineering, a mere subset of developed constructs designated for the remediation of urethral strictures has successfully transitioned into the clinical trial phase, despite the dedication of numerous research decades to this scientific pursuit. The dissemination of our review's findings endeavors to facilitate scientists and clinicians in hastening the development of a clinically efficacious tissue‐engineered urethra, by judiciously harnessing and building upon the extant knowledge derived from preceding studies.

## 1 INTRODUCTION

Urethral stricture is a disease characterized by a multistage fibrotic process that damages the mucosa and may involve a part of the corpus spongiosum. This disease can occur in males of any age, significantly reducing their quality of life and sometimes leading to disability.^1^

Surgery is the primary treatment for strictures. The choice of surgical procedure depends on various factors, including the length and location of the stricture, severity of spongiofibrosis, previous treatment, among others. For extended strictures larger than 2 cm, substitution urethroplasty is the preferred surgical method.^2^ However, the use of patient tissues (e.g., genital or extragenital skin, buccal mucosa) for flaps and grafts can cause morbidity in the donor area and tissue deficiency, necessitating the search for new materials to treat extended and recurrent strictures. Tissue engineering aims to develop new materials suitable for treating patients, but only a few constructs have reached clinical trial stages.^3^, ^4^, ^5^, ^6^, ^7^ Therefore, preclinical trials are still being actively conducted. However, the diversity of data makes it difficult to translate these developments into real clinical practice. Though a few systematic reviews have been conducted previously,^8^, ^9^, ^10^ the most recent one summarized data only up to 2015.^11^ With rapid advancements in the field, we aimed to collate available evidence on the outcomes of preclinical trials of tissue‐engineered urethra in animal models published between 2015 and 2022. Understanding the relationship between scaffold material, cell presence and the success of postoperative outcomes is a necessary step that could greatly enhance the applicability of these findings in a clinical setting.

## 2 MATERIALS AND METHODS

### 2.1 Inclusion criteria

The scope of the research was defined in accordance with PICOS framework: **Participants**: male animal (rabbit, dog, pig). **Intervention**: urethroplasty using tissue‐engineered constructs. **Comparison**: cellular and acellular scaffolds. **Outcomes**: stricture formation and complications (fistula formation, diverticulum, dysuria, urethritis). **Study type**: preclinical studies.

### 2.2 Study characteristics

The manuscript selection criteria included experimental studies that reported the methodology for creating a tissue‐engineered urethra. These studies should have assessed the regenerative potential of the scaffold on an animal model, and evaluated clinical and histological outcomes of treatment. The manuscripts should have been published in English between January 1, 2015 and December 31, 2022.

### 2.3 Search strategy

The search was conducted in the PubMed, Scopus, and PMS databases from September 20–24, 2021 using the keywords: “urethral stricture,” “scaffold,” “tissue engineering urethrae,” and their combinations. A detailed search strategy is outlined in Table S1 in Data S1. An additional search was conducted on September 27, 2022 to identify manuscripts published during the review progress.

### 2.4 Articles selection

**FIGURE 1:**
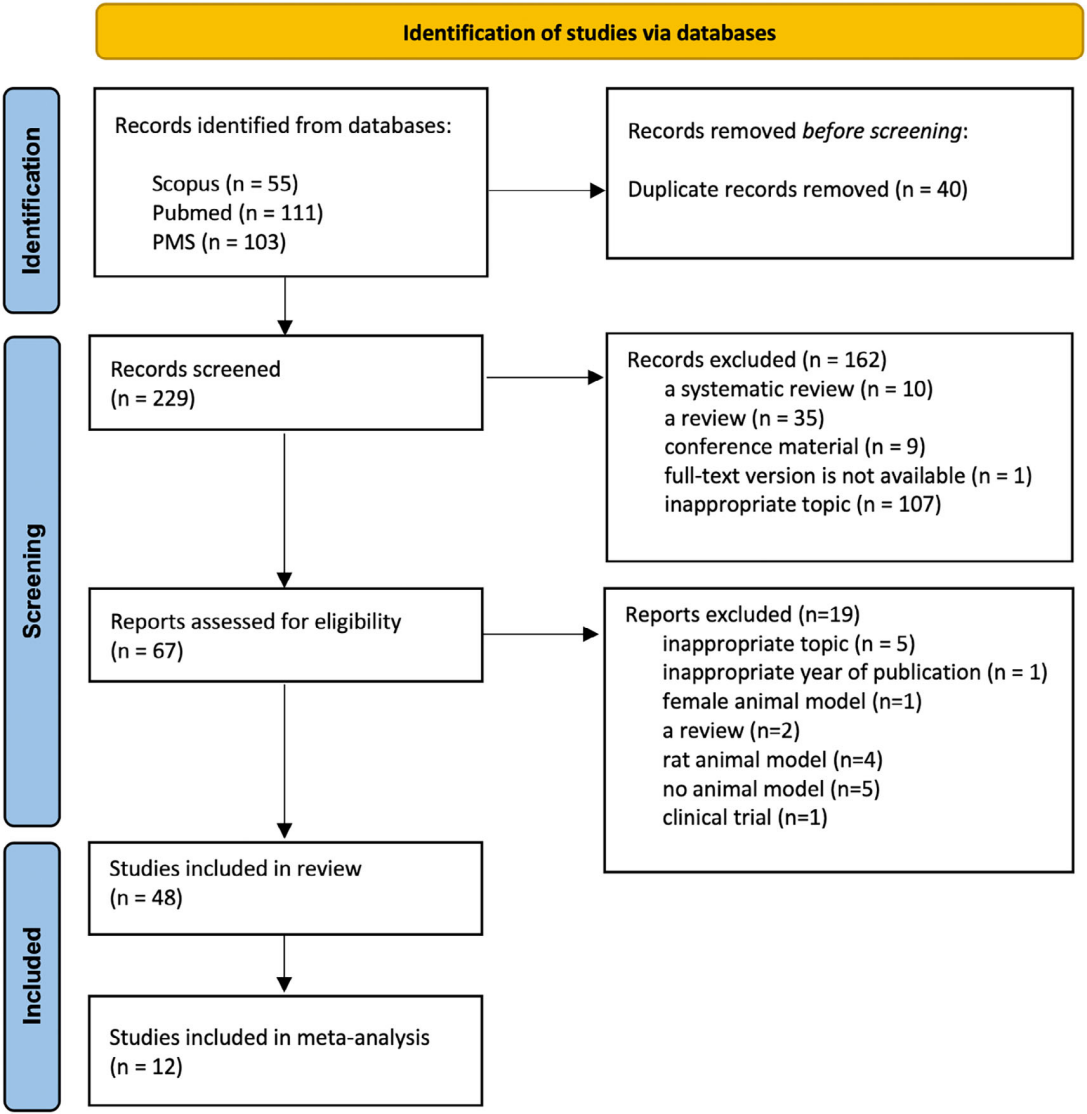
PRISMA flow diagram. PRISMA, Preferred Reporting Items for Systematic Reviews and Meta‐Analyses.

Screening and data extraction were carried out under the Preferred Reporting Items for Systematic Reviews and Meta‐Analyses (PRISMA) checklist.^12^ (Table S2 in Data S1) Conference abstracts, reviews, opinion papers, сase reports, case‐series, letters, and editorials were excluded (Figure 1).

### 2.5 Data extraction

Two reviewers (N.C. and G.S.) independently extracted data from the studies and a third reviewer (A.M.) was involved in case of ambiguous interpretation of data between two extractors. Data on matrix type, cell type (if available), animal model, comparison groups, number of animals per group, defect length, scaffold type (patch or tube), follow‐up period (in weeks), epithelialization time, muscle layer formation time, complications, and outcome detail were extracted.

If number of animals with stricture formation was available from the manuscript, we calculated the success rate (SR) using the following formula: SR = 100% − “failure rate” (%). Here, the 100% refers to the total number of animals in either the experimental or control group within a single study. The failure rate (%) was defined as the number of animals in the same group (experimental or control) that developed a stricture following surgery. We excluded studies involving large female animals and rats due to the inadequacy of their short urethral length for reconstructive procedures.

### 2.6 Strategy for data synthesis

We conducted a comprehensive integrative synthesis of the collated data. The primary objective was to formulate inferences about the correlation between the variety of tissue‐engineered scaffolds and the presence or lack thereof of a cellular component within the tissue‐engineered structure and the subsequent incidence of long‐term postoperative complications and stricture formation. A meta‐analysis was executed exclusively for studies that disclosed the quantity of complications and/or strictures in both the experimental and comparison cohorts. Procedures for data manipulation and subsequent meta‐analysis were performed using R v.4.2 and included libraries such as metafor, meta, and dmetar.^13^, ^14^, ^15^, ^16^

The endpoints were the occurrence of complications and strictures in both the cellular and acellular matrix groups. The odds ratio (OR) was used as the measure of effect size. A random effects model was selected for the meta‐analysis due to the substantial differences in several methodological elements among the studies. Heterogeneity was estimated using the inverse variance method and the restricted maximum likelihood estimator (REML) method was used to determine the dispersion of the effect distribution within the random effects model (τ2). The degree of heterogeneity was assessed by calculating the *Q* statistic and its significance, in addition to the Higgins and Thompson *I* ^2^ statistics. A sensitivity analysis was performed using the leave‐one‐out approach. The effects of different metric types, matrix material, cell type, animal model, and several additional parameters were examined in subgroup analyses and the effect of defect length was evaluated by meta‐regression. The pooled estimate of between‐study heterogeneity was used in the subgroup analysis due to the limited number of studies.^17^ The potential presence of publication bias was assessed visually using funnel plots and, where appropriate, by calculating the Peters test.^18^ The threshold for statistical significance was set at *p* < 0.05.

## 3 RESULTS

### 3.1 Description of the studies

A total of 48 full‐text experimental studies published between 2015 and 2022 were grouped into three main categories based on the scaffold design: (a) аcellular matrices, (b) decellularized matrices, and (c) сell‐seeded (cellular) matrices. Within each section, subcategories were distinguished based on the number of cell types seeded on the matrix including monoculture (only one type of cell culture was seeded) and coculture (two or more types of cell culture were involved).

### 3.2 Participant characteristics

**FIGURE 2:**
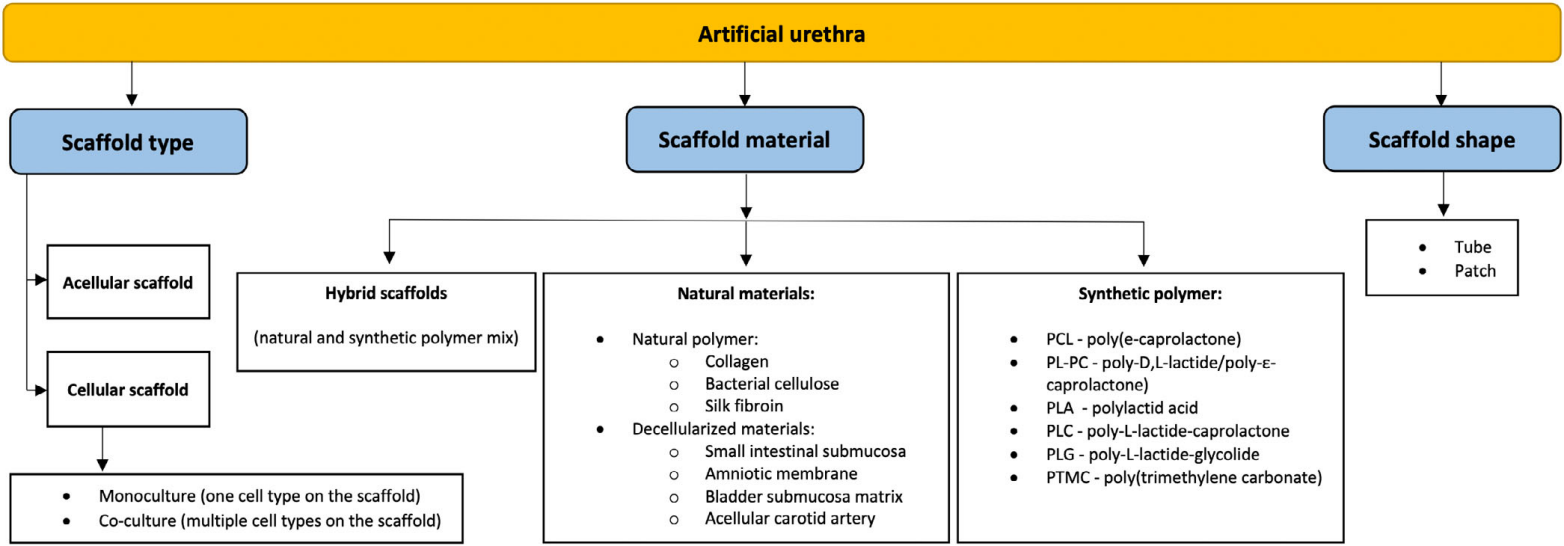
Materials and cells used for tissue‐engineered urethral reconstruction.

Ten studies were conducted in which urethroplasty was performed on male dogs: eight included Beagle breed,^19^, ^20^, ^21^, ^22^, ^23^, ^24^, ^25^, ^26^ in one study breed that was not specified,^27^ and a single study used mongrel dogs.^28^ Male pigs were used in two studies.^29^, ^30^ Chinchilla rabbits were used as experimental animals in three of the studies,^31^, ^32^, ^33^ whereas 33 studies used New Zealand rabbits (Figure 2).

### 3.3 Acellular matrices

During patch scaffold treatment, the maximum length of the defect with no complications and stricture formation was 25 mm, with a follow‐up period of 3 months.^34^ Some studies employing patch scaffold and shorter defect lengths did not attain a 100% success rate in the experimental group.^5^, ^35^, ^36^, ^37^ Tube scaffold treatment displayed a 100% success rate in the treatment of urethral defects measuring 20 mm^38^ and 22 mm^22^ both of which utilized collagen scaffolds. Collagen tubular scaffolds exhibited an 80% success rate at a lengthier follow‐up period on a 20‐mm defect.^39^ When implementing the collagen tubular scaffolds to treat urethral defects of 50 mm in length and follow‐up for 6 months, they demonstrated merely a 60% success rate.^21^, ^26^ Two studies examined scaffolds consisting solely of synthetic polymers, with both reporting stricture formation and associated complications.^5^, ^35^ Six out of 12 studies of natural polymer scaffolds exhibited success without any stricture formation. Among the hybrid scaffold category, four out of five studies resulted in a successful outcome.

Three manuscripts described the use of purely synthetic scaffolds without any modifications. Sartoneva et al. observed no complications in treated with poly‐l‐lactide‐caprolactone (PLC) and poly(trimethylene carbonate) (PTMC) scaffolds, but did note two deaths within the PTMC group.^40^ In two studies, which included polylactid acid (PLA) in the scaffold, the experimental groups failed to achieve a stricture‐free success. In animals without stricture, the scaffolds composed of polylactic acid and glycolic acid, poly(lactic‐co‐glycolic) acid, and l‐lactide‐co‐ε‐caprolactone copolymer PLA/PLG/PLC^35^ or PLA^5^ fully degenerated 6 months after surgery. Among the articles which utilized natural polymer scaffolds, in four studies the authors reported long‐term complications in all groups.^21^, ^26^, ^36^, ^39^ Several studies investigated the combination of natural materials with synthetic ones. Of these, two studies^23^, ^41^ utilized the Collagen l and PLC combination and observed the absence of strictures in their animal experimental groups. Hu et al. developed scaffolds using PLG and gelatin, which were shown to support urothelial cell (UC) proliferation in vitro. However, following urethroplasty, all tissue‐engineered urethras developed fistulas and varying degrees of stenosis in the animal models.^27^ Yang et al. employed a double‐modified bacterial cellulose (BC)/soybean protein isolate (DMBC/SPI) scaffold and observed no strictures or complications 1 month after urethroplasty in the experimental group^42^ (Table 1).

**TABLE 1:** Acellular matrices.

No.	Reference	Matrix	Animal model^a^	Compared groups: number of animals	Size of urethral defect (mm)	Patch or tube	Follow‐up (weeks)	Complete epithelialization (weeks)	Organized smooth muscle (weeks)	Complications	Success rate (%)
**Synthetic polymer scaffolds**											
1	Sartoneva et al.^40^	PLC PTMC	Rabbit	PLC: 15 PTMC: 15 Sham surgery: 2	20	Patch	2, 4, 16	16 16 N/M	N/M N/M N/M	0 2 (died) 0	100 100 100
2	Wan, Zheng, et al.^35^	PLA/PLG/PLC	Rabbit	PLA/PLG/PLC: 6 SIS: 3 Normal group: 3	20	Patch	12, 24	24 – 24	24 – 24	1 3 (1 died) 0	83 33 100
3	Song et al.^5^	PLA	Rabbit	PLA: 6 Sham surgery: 6	15	Patch	4, 12	12 N/M	12 N/M	N/M N/M	83 0
**Natural polymer scaffolds**											
4	Jia et al.^21^	Сollagen	Beagle	Collagen‐CBD‐VEGF: 5 Collagen: 5	50	Tube	24	24 No	– –	2 5	60 0
5	Pinnagoda et al.^39^	Collagen	Rabbit^a^	1 month dCGT: 5 3 months dCGT: 5 6 months dCGT: 5 9 months dCGT: 5	20	Tube	4, 12, 24, 36	– 12 24 36	– – 24 36	4	80
6	Tang et al.^26^	Collagen	Beagle	Collagen‐CBD‐bFGF: 5 Collagen: 5	50	Tube	24	24 –	– –	1 3	80 0
7	Larsson et al.^38^	Collagen	Rabbit	LD graft: 13 HD graft: 9	20	Tube	4, 12, 24, 36, 44	24 12	24 –	2 3	100 100
8	Algarrahi et al.^64^	SF	Rabbit^a^	2 weeks post‐electrocoagulation: 3 1 month group: 3 3 months group: 6 NSC: 3	10	Patch	4, 12	– 4 12 –	– – 12 –	– 0 0 0	– 100 100 100
9	Cao et al.^34^	BSMh; CH; SF	Rabbit	BSMh/SF: 10 CH/SF: 10 SF: 10	25	Patch	4, 12	12 12 –	12 12 12	0 0 N/M	100 – –
10	B. Wang et al.^65^	BC BSM	Rabbit	BC: 6 BSM: 6 BC/BSM: 6	10	Patch	4, 12	No – 12	N/M N/M N/M	1 N/M N/M	0 33 100
11	Fan et al.^66^	SF; BSM; HACC‐BSM	Rabbit	SF/ BSM: 1 SF/HACC‐BSM: 1	15	Patch	4	N/M 4	N/M N/M	N/M N/M	N/M N/M
12	Y. Liu et al.^36^	SF/BSM	Rabbit	SDF‐1α‐aligned SF/3D‐BSM: 12 Aligned SF/3D‐BSM: 12 Nonaligned SF/3D‐BSM: 12	15	Patch	4, 12	12 12 No	12 – –	2 4 10	83 33 33
13	Niu et al.^22^	Collagen/HA	Beagle	Collagen: 6 HA‐collagen: 6	22	Tube	16	N/M N/M	N/M N/M	0 0	100 100
14	Y. Wang et al.^25^	ADM	Beagle	Control group: 7 ADM: 7 ADM‐CBD‐VEGF: 7	30	Patch	24	– 24 24	N/M 24 24	2 2 0	71 100 100
15	Niu et al.^67^	SF SF/HA	Rabbit	SF: 9 SF/HA: 9	20	Tube	6, 14	14 14	– 14	N/M N/M	– –
**Hybrid scaffolds**											
16	Lv, Li, et al.^68^	Keratin/SF/gelatin; CPO	Rabbit	Films + CPO: 6 Films alone: 6 SIS: 6	15	Patch	24	24 – 24	24 24 24	0 6 0	100 N/M 100
17	K. Zhang et al.^23^	Collagen l /P(LLA‐CL)	Beagle	Сonjugated nanofibrous: 2 Nanoyarn: 4 (ICG‐001) nanoyarn: 4	20	Patch	6, 12	Nо Nо 12	N/M N/M N/M	2 1 1	0 50 100
18	Hu et al.^27^	PLG, gelatin	Dog	PLG: 6 PLG/gelatin: 6	20	Tube	4, 12	No No	No No	2 1	0 0
19	L. Wang et al.^41^	Collagen l/PLC	Rabbit	Non‐ADSC‐exos: 3 ADSC‐exos nanoyarn: 3	20	Patch	4	No 4	No 4	N/M 0	– 100
20	Yang et al.^42^	DMBC/SPI	Rabbit	DMBC/SPI: 6 BC: 6 ADMG: 6	22	Patch	2, 4	4 No No	4 N/M N/M	0 0 N/M	100 – N/M

*Note*: ICG‐001, Wnt signaling inhibitor; –, due to the lack of complete information in the article, it was not possible to perform the calculation; N/M (not mentioned), the manuscript lacks the specific information required for our analysis; No, cell layers have not formed.

Abbreviations: ADM, acellular dermal matrix; ADSC‐exos, adipose‐derived stromal cells' exosomes; BC, bacterial cellulose; BSM, bladder submucosa matrix; BSMh, bladder submucosa matrix hydrogel; CBD‐VEGF, collagen‐binding VEGF; CH, collagen type I hydrogel; CPO, calcium peroxide; dCGT, double‐layered collagen gel tubes; DMBC/SPI, double‐modified bacterial cellulose/soybean protein isolate; HA, hyaluronic acid; HACC, 2‐hydroxypropyltrimethyl ammonium chloride chitosan; HD graft, high fiber density collagen graft; LD graft, low fiber density collagen graft; NSC, nonsurgical control; PLA, polylactid acid; PLC, poly‐l‐lactide‐caprolactone; PLG, poly‐l‐lactide‐glycolide; PTMC, poly(trimethylene carbonate); SDF‐1α, stromal cell‐derived factor‐1 alpha; SF, silk fibroin; SIS, small intestinal submucosa.

^a^ A chronic urethral defect created by a person a few weeks before urethroplasty.

### 3.4 Decellularized matrices seeded with cells

#### 3.4.1 Small intestinal submucosa (SIS)

Although SIS was the predominantly utilized matrix in the reviewed studies, only one^43^ of the four studies did not exhibit postoperative stricture formation. Guo et al. employed SIS supplemented with oral keratinocytes (OK) and fibroblasts transfected with tissue inhibitor of metalloproteinases 1 (FB‐TIMP‐1 siRNA) to fabricate the scaffold; nonetheless, complete relapse‐free success was not achieved.^44^ Liu et al.^45^ and Amesty et al.^46^ also reported unsuccessful outcomes in the experimental group. In contrast, in the experimental group strictures or fistulas did not develop when the SIS surface was modified with 5% peracetic acid (PAA) in a single study.^43^

#### 3.4.2 Amniotic membrane (AM)

AM has been utilized as a tube scaffold in three studies.^28^, ^47^, ^48^ In two cases^28^, ^48^ the scaffold resulted in 100% success rate. Chen et al. employed decellularized human amniotic scaffold seeded with allogenic bone marrow mesenchymal cells (BMSC) and/or endothelial progenitor cells (EPC) to simulate urethral tissue structure.^28^ Two months after surgery, urethrography demonstrated a urethral caliber similar to that of healthy urethras in both the AM + BMSC + EPC and AM + EPC groups. D. Zhang et al. reported newly formed epithelium without inflammatory infiltration covered the urethra in both fresh epithelial cell sheet (SEC) with AM and cryo‐SEC‐AM groups 1 month after urethroplasty.^48^ In the study by Hariastawa et al., where dried AM scaffolds and adipose‐derived stromal cells (ADSCs) were used, high animal mortality was recorded in both groups. The success rate could not be calculated due to insufficient reporting on the causes of rabbit death and the replacement of deceased rabbits with new ones.^47^

#### 3.4.3 Bladder submucosa matrix (BSM)

Two studies of BSM demonstrated a lack of stricture development in the treatment of a urethral defect measuring 20 mm and 30 mm, respectively, through the implementation of both patch^49^ and tube^50^ scaffolds.

#### 3.4.4 Acellular carotid artery (ACA)

A study conducted by Zhong et al. detailed the application of an ACA tube scaffold for the purpose of mending a 30‐mm defect within the Beagle model. This investigation yielded a 50% success rate in the unseeded ACA group and a 100% success rate in the ADSC‐seeded scaffold group^24^ (Table 2).

**TABLE 2:** Decellularized matrices.

Type	No.	Reference	Matrix	Type of autologous cells	Animal model	Compared groups: number of animals	Size of urethral defect (mm)	Patch or tube	Follow‐up (weeks)	Complete epithelialization (weeks)	Organized smooth muscle (weeks)	Complications	Success rate (%)
Matrices with monoculture of cells	1	Chun et al.^49^	BSM	aUT^a^	Rabbit^d^	Normal control (sham): 5 Urethral stricture (simple urethrotomy): 5 BSM: 5 BSM + UT: 5	20	Patch	4, 8, 12	– No No 12	– No No 12	0 0 0 0	100 0 100 100
	2	L. Zhang et al.^43^	SIS	aUC	Rabbit	5% PAA treated SIS + UC: 6 Untreated SIS + UC: 6 5% PAA treated SIS: 6	15	Patch	24	24 – No	24 – –	0 3 0	100 100 0
	3	Y. Liu et al.^45^	SIS	aUSC	Rabbit	SIS + USC: 12 SIS: 12	20	Patch	2, 3, 4, 12	2 12	12 –	N/M N/M	92 0
	4	Hariastawa et al.^47^	AM	aADSC	Rabbit	Control group: 8 (3 died) AM: 8 (5 + 3 died) AM + ADSC: 8	10	Tube	4	– – –	– – –	0 4 2	– – –
	5	Zhong et al.^24^	ACA	aADSC	Beagle	ACA + ADSC: 6 ACA: 6	30	Tube	4, 12	4 No	4 No	0 2	100 50
	6	Amesty et al.^46^	SIS	aUC	Rabbit^d^	Urethral defect unrepaired: 2 SIS: 10 SIS + UC: 10	18	Patch	4	No No 4	No No N/M	No 10 5	No 0 100
	7	D. Zhang et al.^48^	AM	aOK	Rabbit	Sham operation: 3 Urethrotomy: 3 AM: 3 Fresh SEC‐AM + OK: 3 Cryo‐SEC‐AM + OK: UR^a^	20	Tube	4	N/M N/M N/M 4 4	N/M N/M N/M N/M N/M	N/M N/M N/M 0 0	0 N/M N/M 100 100
Matrices with сoculture of cells	8	Guo et al.^44^	SIS	aOK FB	Rabbit	SIS: 8 (1 dead) SIS + OK: 8 SIS + OK + FB^b^: 8	20	Patch	4, 24	24 4 4	No 24 24	N/M N/M N/M	71 75 88
	9	Li et al.^50^	BSM	aEPC^c^ aUC aSMC	Rabbit	BSM + UC + SMC + EPC^c^: 4 BSM + UC + SMC + EPC: 4 BSM: 2	30	Tube	8	8 No –	8 N/M –	– – –	100 100 –
	10	Chen et al.^28^	AM	aBMSC aEPC	Dog	Normal group: 5 AM + BMSC + EPC: 5 AM + EPC: 5 AM + BMSC: 5 Blank AM: 5 Sham operated group: 5	30	Tube	4, 8	8 8 8 8 No No	N/M 8 N/M N/M N/M N/M	0 0 1 N/M N/M N/M	100 100 100 – 0 0

*Note*: –, due to the lack of complete information in the article, it was not possible to perform the calculation; a, allogeneic or autologous cells; h, human cells; N/M (not mentioned), the manuscript lacks the specific information required for our analysis; No, cell layers have not formed.

Abbreviations: ACA, acellular carotid artery; ADSC, adipose‐derived stromal cells; AM, amniotic membrane; BMSC, bone marrow stromal cells; BSM, bladder submucosa matrix; EPC, endothelial progenitor cells; FB, fibroblasts; OK, oral keratinocytes; PAA, peracetic acid; SEC, epithelial cell sheet; SIS, small intestinal submucosa; SMC, smooth muscle cells; UC, urothelial cells; USC, urine‐derived stem cells.

^a^ Unclear results (UR); the article incorrectly indicates the number of animals and study groups.

^b^ Fibroblast transfected with tissue inhibitor of metalloproteinase‐1 small interfering RNA.

^c^ EPC were transfected with lentiviral vectors expressing the antibiotic peptide LL37.

^d^ A chronic urethral defect created by a person a few weeks before urethroplasty.

### 3.5 Cell‐seeded matrices: cell types used in urethral tissue engineering

#### 3.5.1 Matrices with a monoculture of cells

The two studies utilizing tubular scaffolds were successful in achieving a stricture‐free outcome with urethral reconstruction of defects measuring 15 mm^51^ and 20 mm,^52^ respectively. Stricture‐free success was not achieved in only two studies.^53^, ^54^ In three studies, urethral defects were created several weeks prior to reconstructive urethroplasty.^29^, ^30^, ^54^ Follow‐up periods ranged from 1 to 24 weeks, with the longest follow‐up period resulting in absence of stricture in three studies.^30^, ^51^, ^55^

#### 3.5.2 Matrices with coculture of cells

The present literature encompasses five studies that report the utilization of several cell cultures for matrix seeding: synthetic polymer matrix^19^, ^56^ and hybrid matrix.^57^ Two studies describe innovative approaches including the use of hemostatic commercial gelatin sponge “Spongostan”^31^ as a matrix and scaffold‐free technology utilizing cell sheet.^20^

#### 3.5.3 UC

UC obtained from bladder biopsy have been most commonly utilized for creating cellular scaffolds. Successful outcomes have been achieved through the use of collagen scaffolds^29^, ^30^ in conjunction with monocultures of UC, as well as combined matrices^52^, ^57^ of natural and synthetic polymers. Synthetic scaffolds^19^, ^56^ combined with cocultures of smooth muscle cells (SMC) and UC have also shown promise in animal experiments. The use of SIS with UC has not yielded consistent results. While Zhang et al.^43^ reported stricture‐free success, Liu et al.^45^ was successful in 50% of cases. The use of the complex BSM scaffold in combination with three cell cultures reports no stricture formation.^50^ Amesty et al. have reported on the successful inoculation of commercial SIS with UC harvested from bladder washes. After 4 weeks, no cases of stricture or diverticula were observed in any of the animals in the experimental group.^46^ Aufderklamm et al. utilized xenogeneic transplantation of human cells in their animal experiments and found no instances of stricture or other complications in any of the four minipigs after 2 weeks.^29^

#### 3.5.4 Stem cells from urine

Liu et al. exhibited the differentiation of urine‐derived autologous stem cells (USC) into UC and SMC in an in vivo setting. The experimental group manifested complete epithelial cell layers within 2 weeks. After a period of 3 months, urethrogram results indicated that only one rabbit in the SIS + USC group exhibited stricture, while all subjects in the control group developed it.^45^

#### 3.5.5 Urethral tissue

Chun et al. proposed a method that shorten the duration of scaffold creation by utilizing autologous urethral tissue in conjunction with an acellular BSM. Following 12 weeks of analysis, urethrography results displayed that the average urethral width in both the BSM and BSM/autologous urethral tissue groups was comparable to the normal urethra.^49^

#### 3.5.6 ADSCs

Evaluating ADSC use in urethral tissue engineering on animal models yielded favorable outcomes in four^20^, ^24^, ^55^, ^58^ of the six studies considered in this review.

#### 3.5.7 OK

Huang et al. utilized OK as seeding cells on 3D‐porous BC scaffolds. After a period of 3 months, the group treated with 3D‐porous BC + OK demonstrate a wide calibers of the urethra with an intact epithelial lining.^59^ Guo et al. employed both OK and fibroblast transfected with tissue inhibitor of metalloproteinase‐1 small interfering RNA (TIMP‐1 siRNA) within their scaffold. The authors observed that epithelial cell layers formed in the OK + SIS and SIS + OK + FB groups, while the SIS alone group exhibited chronic inflammation and fibrosis.^44^ Yudintceva et al. compared a synthetic two‐layer polycaprolactone/poly(lactic‐co‐glycolic) acid scaffold with autologous OK and autologous buccal flap (BF). The authors noted that the scaffold fully degraded, with the urethral lumen and integrity in experimental groups being comparable to intact controls.^32^

#### 3.5.8 Skin keratinocytes

In Rogovaya et al.'s 2015 study, a connective tissue equivalent was prepared by culturing fibroblasts in collagen gel on the surface of a Spongostan™ and then seeded with using rabbit skin keratinocytes. After transplantation of this living skin equivalent (LSE) into de‐epithelialized urethra, keratinocytes were observed to invade the damaged tissue and develop as urothelium. The reconstructed urethra exhibited morphology corresponding to stratified squamous epithelium.^31^ The use of rabbit ear skin as a cell source may have contributed to differences between the restored and native urothelium. In 2021, Zhang et al. carried out the first study that successfully preserved the viability of an epithelial cell sheet combined with a human acellular AM through cryopreservation. The percentage of proliferating keratinocytes was comparable in both the cryopreservation and control groups, suggesting promising prospects for tissue‐engineered construct cryopreservation.^48^

#### 3.5.9 Mesothelial cells (MC)

In 2017, Jiang et al. proposed the use of autologous granulation tissue (GT) as a matrix. MC were obtained via biopsy from rabbit omentum and were then planted on a tubular matrix of GT. Examination of unseeded grafts using macroscopic analysis revealed a shrinkage across the whole graft. In contrast, the GT + MC graft group demonstrated less significant shrinkage, an absence of fibrosis, and a wide urethral lumen visible on urethrogram imaging. Newly formed urothelium was observed to have completely replaced the original mesothelium.^51^

#### 3.5.10 Amniotic mesenchymal cells

Lv et al. utilized PLA with poly(ethylene glycol) (PEG) scaffolds seeded with human amniotic mesenchymal cells (AMSC). Post‐surgical evaluation using urethrography demonstrated an absence of strictures within the experimental AMSC + PLA/PEG group, unlike the two comparator groups. The PLA/PEG cohort exhibited no formation of an epithelia layer, with only 50% of the cell‐free matrices degraded after 4 weeks. In contrast, the AMSC‐seeded PLA/PEG group exhibited the presence of multilayered urothelium.^60^

#### 3.5.11 Bone marrow‐derived mesenchymal stromal cells (MSC)

Yudintceva et al. conducted a study demonstrating the feasibility of MSC differentiation into urothelium. The researchers utilized a bilayered synthetic poly‐d,l‐lactide/poly‐ε‐caprolactone scaffold seeded with allogeneic BMSC. A follow‐up period of 12 weeks post‐urethroplasty revealed no complications across all participant groups, as well as nanoparticle‐labeled MSCs being identified within the urothelial and muscular layers of the reconstructed urethra.^33^

#### 3.5.12 EPC

Three distinct cell types, namely EPC, UC, and SMC, were employed to seed a tubular acellular bladder matrix. EPC were transfected with lentiviral vectors that expressed the antibiotic peptide LL37. In the experimental group where BSM was subjected to seeding with cells that were transfected with LL37, fully formed layers of transitional epithelium and well‐developed smooth muscle tissue were observed. On the other hand, the group without transfection exhibited only a partially formed epithelial layer and inadequate vascularity.^50^

Chen et al. aimed to investigate the efficacy of transplantation of BMSC and/or EPC. The group subjected to AM + BMSC + EPC did not report any untoward postoperative clinical manifestations. The AM + BMSC + EPC and the AM + EPC cohort revealed complete coverage of the inner scaffold surface with stratified columnar epithelium.^28^

#### 3.5.13 SMC

In the study conducted by Niu et al.,^19^ the amphiphilic scaffold was composed of hydrophilic PEG and hydrophobic poly(ε‐caprolactone), onto which autologous UC and SMC were seeded. The same group of researchers created a scaffold with a hierarchical architecture of alternating block polyurethane (PU‐alt) nanofibers, comprising PEG and PCL and employed the same cell type combination.^56^ Scaffolds degradation in both studies was observed within 30 days, with minimal differentiation between the scaffold and native tissue. The PCL scaffold group in the first study^19^ showed irregularly arranged SMC, while the second study^56^ showed no expression of muscle tissue markers in the urethral wall. In a study by G. Liu et al., scaffolds with varying ratios of PLA and gelatin were evaluated for their effect on OK and SMC cell adhesion and proliferation. Enhanced hydrophilicity of the PLA/gelatin scaffold favored cell growth over PLA. The PLA/gelatin scaffold with a ratio of 50:50 did not promote directed growth of cells, whereas OK and SMC were evenly distributed in the PLA/gelatin scaffold with a ratio of 75:25.^57^ Wang et al. coated a polyurethane‐urea (PUU) fiber membrane with collagen (cPUU), which led to increased epithelial thickness compared to the PUU group.^37^ All animal groups developed fistulas.

#### 3.5.14 Cell sheet technology

Zhou et al. conducted a study aimed at developing a bionic urethra using cell sheet technology for the first time. ADSC, OK, and oral FB were utilized to prepare the cell layers. The ADSCs were pre‐differentiated into myoblasts. To emulate the histological structure of the native urethra, they assembled two sheets of epithelial cells, one layer of fibroblasts, and two layers of differentiated myoblasts. The resulting three‐layer urethra was then wrapped around a silicone tube and labeled through ultrasmall super‐paramagnetic iron oxide (USPIO). To trigger vascularization, the construct was implanted subcutaneously in the groin of beagles and left for 3 weeks. After implantation, the cell sheets approached closer together while immunofluorescence analysis demonstrated a significant increase in blood vessel density. Three months after the urethroplasty, urethrograms indicated that all dogs in the tissue‐engineered bionic urethra group and the autologous buccal mucosa group had no strictures. USPIO‐labeled cells were visible in the transplanted area, affirming their viability over the period. Histological examination of fragments of the bionic urethra revealed its continued composition of three layers: epithelial, fibrous, and muscular^20^ (Table 3).

**TABLE 3:** Cell‐seeded matrices.

No.	Reference	Matrix	Type of autologous cells	Animal model^k^	Compared groups: number of animals	Size of urethral defect (mm)	Patch or tube	Follow‐up (weeks)	Complete epithelialization (weeks)	Organized smooth muscle (weeks)	Complications	Success rate (%)
**Matrices with monoculture of cells**												
Synthetic polymers scaffolds												
1	D. Wang et al.^53^	PLA	aADSC	Rabbit	PLA + ADSC: 12 PLA: 12	10	Patch	4, 6	4 No	6 N/M	2^a^ 4	75 50
2	Lv, Guo, et al.^60^	PLA/PEG	hAMSC	Rabbit	PLA/PEG + AMSC: 9 PLA/PEG: 9 Sham surgery: 9	20	Patch	4, 8, 12	12 No –	12 N/M –	0 – –	100 94 28
3	Wan et al.^55^	PLA/PCL/PLG^c^ PLA/PLG/PCL^d^	aADSC	Rabbit	Hypoxia‐preconditioned: 6 Normoxia‐preconditioned: 6 Intact urethra: 3	20	Patch	12, 24	24 – –	24 – –	2 5 0	100 100 100
4	Yudintceva, Nashchekina, Shevtsov, et al.^32^	PLC/PLG	aOK	Rabbit	PLC/PLG + OK: 4 Buccal flap: 4 Intact animals: 2	7	Patch	12	12 12 12	12 12 12	0 0 0	100 100 100
5	Yudintceva, Nashchekina, Mikhailova, et al.^33^	PL‐PC	aBMSC	Rabbit	PL‐PC + BMSC: 9 BMG: 9 Intact rabbit: 1	7	Patch	4, 8, 12	12 12 –	12 12 –	0 0 0	100 100 100
Natural polymer scaffolds												
6	Huang et al.^59^	BC	aOK	Rabbit	BC: 10 3D porous BC: 10 3D porous BC + OK: 10	20	Patch	4, 12	12 12 4	12 12 12	N/M N/M 0	0 0 100
7	Aufderklamm et al.^29^	Collagen l (CCC)	hUC	Pigs^k^	CCC + UC (luminal): 2 CCC + UC (anti‐luminal): 2	10–15	Patch	2	– –	N/M N/M	1 0	100 100
8	Tian et al.^54^	SF	aADSC	Rabbit^k^	Control group: 13 SF group: 13 SF + ADSC: 13	25	Patch	2, 4, 6	No 6 4	No 4 4	10 3 2	23 77 85
9	Sievert et al.^30^	Collagen	aUC	Pigs^k^	1 week: 2 2 weeks: 2 4 weeks: 2 24 weeks group: 2	20	Patch	1, 2, 4, 24	N/M N/M N/M N/M	N/M N/M N/M N/M	0 0 0 0	100 100 100 100
10	Zhu et al.^58^	BC	aADSC	Rabbit	Сut‐and‐suture control: 6 BC + ADSC: 6 BC + ADSC‐FGFR2: 6	15	Patch	4, 12	12 12 4	12 12 4	5 (1 died) 4 (1 died) 2	83 83 100
Hybrid scaffolds												
11	K. Zhang et al.^52^	Collagen; PLC	aUC	Rabbit	Col/PLC + UC: 6 ICG‐001 Col/PLC + UC: 6	20	Tube	12	– 12	– 12	1 0	17 100
12	C. Wang et al.^37^	Collagen/PUU	aSMC	Rabbit	Collagen/PUU‐fibrous hydrogel: 8 PUU scaffold: 8 Sutured directly: 8	15	Patch	12	12 – 12	12 – –	1 3 4	88 63 0
Extra^k^												
13	Jiang et al.^51^	aGT	aMC	Rabbit	GT + MC: 9 GT: 9	15	Tube	4, 8, 24	4 8	24 No	0 –^b^	100 –
**Matrices with сoculture of cells**												
Synthetic polymers scaffolds												
14	Niu, Liu, Chen, et al.^19^	PU‐ran PCL	aSMC aUC	Beagle	Autograft: 8 PU‐ran + SMC + UC: 8 PCL + SMC + UC: 8 Blank control groups: 8	22	Tube	1, 2, 4^7^, 8^8^; 12	8^d^ 8^d^ – No	8^d^ 8^d^ – –	N/M N/M N/M N/M	100 100 0 N/M
15	Niu, Liu, Fu, et al.^56^	PU‐alt^f^	aSMC aUC	Rabbit	Autograft: 3 PU‐alt + UC + SMC: 5 PCL + UC + SMC: 3 Blank control groups: 2	22	Tube	4^g^, 8^h^, 12, 14^j^	12 12 No No	12 12 No No	– 0 – –	100 100 0 0
Hybrid scaffolds												
16	G. Liu et al.^57^	PLA/gelatin	aSMC aUC	Rabbit	Autograft: 3 PLA/gelatin + UC + SMC: 3 PLA: 3 Blank control group: 3	22	Tube	12	12 12 No –	12 12 No –	N/M N/M N/M –	100 100 0 –
Extra^k^												
17	Rogovaya et al.^31^	LSE^e^	aOK aFB	Rabbit	LSE + OK + FB: 11 De‐epithelialized urethra: 3 Сellfree Spongostan™: 3	10–15	Tube	2, 4^g^, 6^i^, 12	6 N/M N/M	N/M N/M N/M	0 3 3	100 N/M N/M
18	Zhou et al.^20^	No (cell sheet)	aADSC aOK aFB	Beagle	USPIO‐labeled cell sheet: 6 Unlabeled cell sheet: 6 SIS: 6 aBuccal mucosa: 6	20	Tube	12	12 12 No 12	– – No –	0 0 2 0	100 100 33 100

*Note*: –, due to the lack of complete information in the article, it was not possible to perform the calculation; a, allogeneic or autologous cells; h, human cells; ICG‐001, Wnt signaling inhibitor; N/M (not mentioned), the manuscript lacks the specific information required for our analysis; No, cell layers have not formed.

Abbreviations: ADSC, adipose‐derived stromal cells; AMSC, amniotic mesenchymal cells; BC, bacterial cellulose; BMG, buccal mucosa graft; BMSC, bone marrow stromal cells; CCC, bovine collagen type I‐based cell carriers; FB, fibroblasts; FGFR2, fibroblast growth factor receptor 2; GT, granulation tissue; LSE, living skin equivalent; MC, mesothelial cells; OK, oral keratinocytes; PCL, poly(ε‐caprolactone); PEG, poly(ethylene glycol); PLA, polylactid acid; PLC, poly‐l‐lactide‐caprolactone; PLG, poly‐l‐lactide‐glycolide; PL‐PC, poly‐d,l‐lactide/poly‐ε‐caprolactone; PU‐alt, alternating block polyurethane; PU‐ran, polyurethane block copolymer; PUU, polyurethane‐urea; SF, silk fibroin; SMC, smooth muscle cells; UC, urothelial cells; USPIO, ultrasmall super‐paramagnetic iron oxide.

^a^ In a study by D. Wang et al.,^53^ scaffold contraction was observed in 2 of 12 rabbits in the PLA + aADSCs group, as well as in 4 of 12 rabbits in the cell‐free PLA group.

^b^ In the study by Jiang et al.,^51^ scaffold contraction was observed only in the group of cell‐free matrix from granulation tissue; the number of individuals was not mentioned.

^c^ Microporous inner layer: poly‐l‐lactic acid (PLA)/poly‐l‐lactide‐glycolide (PLG)/poly‐l‐lactide‐caprolactone (PLC) blend (ratio of 20:20:60).

^d^ Macroporous side: PLA/PLG/poly caprolactone (PCL) blend (ratio of 30:40:30).

^e^ Hemostatic commercial sponge “Spongostan.”

^f^ Chemical structure of PU‐alt based on PEG (poly(ethylene glycol) and PCL (poly‐ε‐caprolactone)).

^g^ 30 days.

^h^ 60 days.

^i^ 45 days.

^j^ 100 days.

^k^ A chronic urethral defect created by a person a few weeks before urethroplasty.

### 3.6 Meta‐analysis

#### 3.6.1 Complications

**FIGURE 3:**
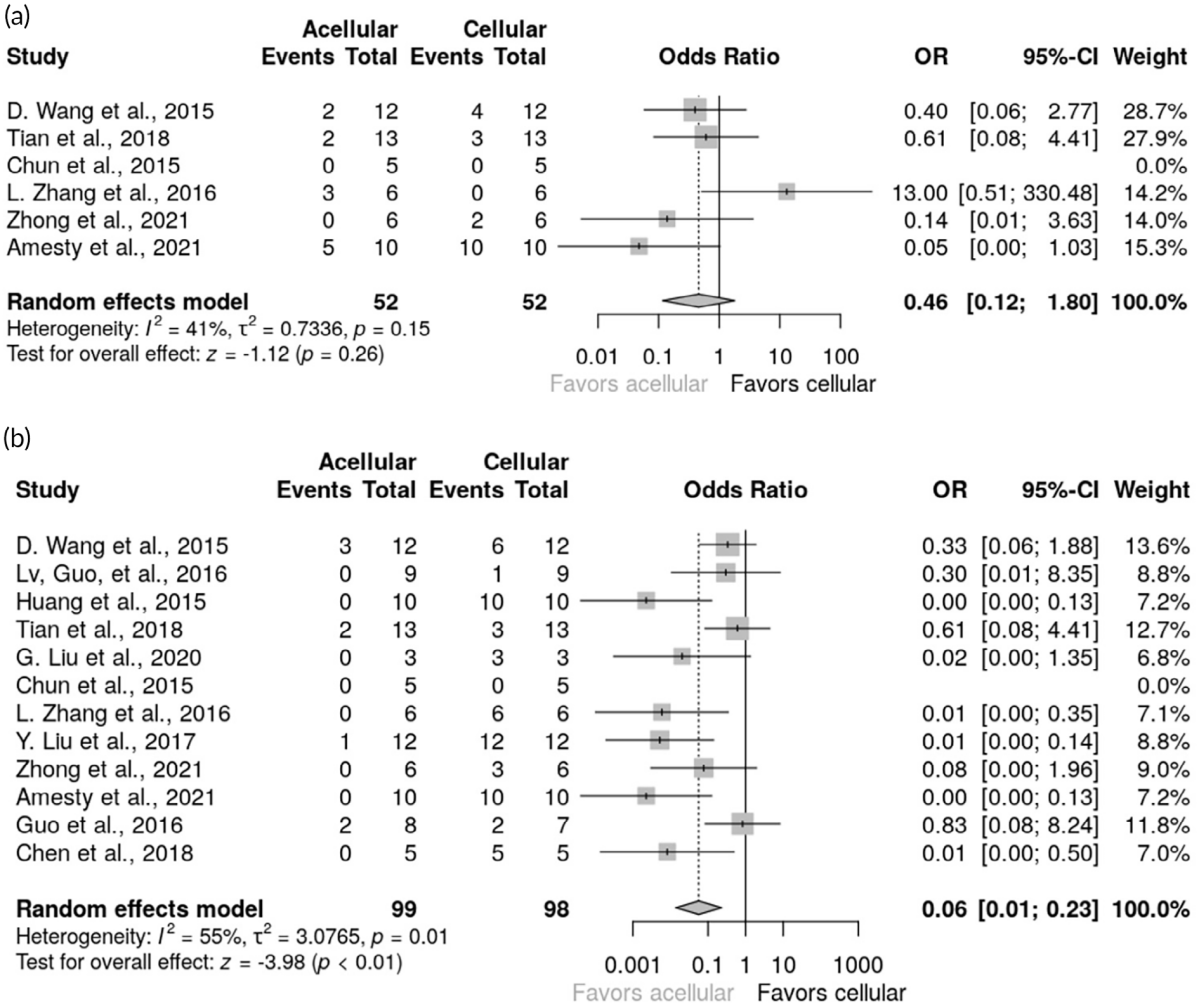
(a) Forest plot of the risk of complications depending on the type of matrix (acellular, cellular). (b) Forest plot of the risk of strictures depending on the type of matrix (acellular, cellular).

In evaluating the risk of complications, the evidence did not find a statistically significant advantage for any of the methodologies used (OR 0.46 [95% CI 0.12; 1.80]). Similarly, no noticeable benefits were observed for any of the matrix types when comparing disparities in complications. Given the small sample sizes used in these studies, detecting subtle changes in odds proved challenging. Sensitivity analysis did not show any significant alterations in the effect size or heterogeneity following the exclusion of individual studies. According to the funnel plot and Peters' test results (*p* = 0.53) there was no indication of potential publication bias (Figure 3).

##### Subgroup analysis

The subsequent analysis aimed to evaluate the potential impact of factors such as the type of matrix, the variety of cells, the animal model, and the kind of scaffold. However, none of these effect modifiers significantly swayed the overall effect within the subgroups. This subgroup analysis was notably constrained by the limited number of trials, leading to insufficient statistical power. The length of the defect was incorporated as a covariate within a univariate meta‐regression model, yet it demonstrated no significant effect on the risk of complications, with β1 = −0.06 [−0.31; 0.19] and *p* = 0.62. The degree of explained variance by the defect length was negligible, amounting to zero (*R* ^2^ = 0) (Figures S1–S7 in Data S1).

#### 3.6.2 Strictures

The overall effect demonstrated a significant benefit in favor of acellular matrices (OR 0.06 [95% CI 0.01; 0.23]). Sensitivity analyses corroborated the robustness of the overall significant effect, as indicated by the leave‐one‐out procedure. Results from the funnel plot and Peters' test (*p* = 0.76) revealed no potential presence of publication bias (Figure 3).

##### Subgroup analysis

The set of potential effect modifiers mirrored those associated with the endpoint of complications. Given the considerable number of subgroups, it would be apt to acknowledge the observed trends. Decellularized matrices and synthetic polymer subgroups maintained a consistent and significant trend with regards to the overall effect. Of all cell types, the “UC” subgroup exhibited the highest incidence of strictures, with an OR approaching 0 [95% CI 0; 0.06]. While various effects (albeit insignificant) were identified across animal model and scaffold type subgroups, acellular matrices proved beneficial in all instances. The length of the defect did not present a significant influence in the meta‐regression analysis, with β1 equating to −0.02 [−0.28; 0.23] and a *p*‐value of 0.86 (Figures S8–S14 in Data S1).

## 4 DISCUSSION

This systematic review collates evidence from preclinical trials of tissue‐engineered urethras. The academic community's interest in natural and synthetic polymer‐based cell‐free matrices can be explained by the absence of labor‐intensive cellular cultivation. The predominant polymers utilized in the fabrication of urethral scaffolds include poly‐l‐lactic acid, polycaprolactone, and their composites with each other as well as with natural polymers such as collagen. However, preclinical trials with non‐modified synthetic polymer scaffolds suggest the low effectiveness of using such scaffolds in urethral tissue engineering. Research involving synthetic scaffolds revealed the emergence of postoperative strictures and additional complications. Given the absence of specific enzymes in the human body capable of degrading synthetic polymers, it is chronic inflammation that result in the excessive production of connective tissue and the formation of postoperative strictures. Our findings indicate that the formation of the epithelial layer on cell‐free matrices requires a significantly extended duration compared to scaffolds that had been seeded with cells. This process may be attributed to the ability of cells on pre‐seeded scaffolds to synthesize extracellular matrix on the scaffold's surface. This pre‐synthesized matrix can subsequently enhance the proliferation of the host's own cells on the scaffold following its implantation (Table 4).

**TABLE 4:** Mechanisms of biointegration of different types of materials.

Materials	Mechanisms	
	Biodegradation and inflammation	Regeneration
**Synthetic polymers**		
Poly‐l‐lactic acid, polycaprolactone, poly(trimethylene carbonate), polyurethane‐urea	Synthetic polymer materials are subject to nonspecific resorption. During incomplete phagocytosis, macrophages and giant multinucleated cells regulate tissue response and can form granuloma. Activated fibroblasts overproduce collagen and other connective tissue proteins, culminating in the development of a dense capsule encircling the implant. Degradation of some polymers (e.g., PLA) reduces pH levels, which subsequently enhances the activity of TGFbeta1, a principal mediator of fibrosis.	Synthetic materials do not expedite regeneration; rather, they facilitate optimal mechanical conditions for biointegration of artificial tissue. These materials may stimulate matrix remodeling through the fibrotic activation of fibroblasts.
**Decellularized tissue**		
Bladder submucosa matrix, amniotic membrane, acellular carotid artery, small intestinal submucosa	Decellularized materials are subject to resorption through the action of specific enzymes, such as collagenases. The resorption of the implant is followed by replacement with the host's own tissue. The duration of degradation is typically brief and uncontrolled. It must be noted that materials sourced from cadavers (e.g., acellular carotid artery) may be damaged by calcification. Inflammation may be caused by either remnants of cellular components or chemicals employed in the decellularization process.	Decellularized materials maintain the structural scaffolding for blood vessels and parenchymal cells. Preserved signaling enhance the recellularization by host cells and direct regeneration toward the reconstruction of the original histoarchitectural configuration. As degradation occurs, the implanted material becomes a provisional matrix that supports de novo tissue formation.
**Natural polymers**		
Bacterial cellulose	The specific mechanism for the resorption of bacterial cellulose involves the hydrolytic cleavage of glycosidic bonds within cellulose chains by cellulase enzymes. Since cellulase is not synthesized in the human body, phagocytosis remains the sole resorption mechanism of bacterial cellulose implants. Given that macrophages are unable to directly break down cellulose into glucose, the resorption process for such materials can extend over months or even years.	Bacterial cellulose exhibits high biocompatibility and typically elicits a minimal inflammatory response following implantation. It establishes mechanical conditions conducive to the restoration of urethral function, without impeding epithelialization or the recovery of barrier function.
Collagen	Collagen, a natural protein in the human body, exhibits exceptional compatibility with human tissues, thereby diminishing the likelihood of adverse immune responses. Collagen is susceptible to degradation through the body's inherent metabolic processes. This degradation permits the material to diminish at a pace commensurate with the development of new tissue, ultimately resulting in the complete absorption of the collagen with no residual foreign substances.	Contains Arg‐Gly‐Asp (RGD) tripeptide sequences that may facilitate the adhesion, proliferation, and migration of various cell types, including epithelial, endothelial, and fibroblast cells. Collagen fibrous materials facilitate rapid migration of cells and can become provisional matrix when resorbed by host's macrophages and fibroblasts.
Silk fibroin	Silk fibroin undergoes resorption through hydrolysis and enzymatic cleavage in the human body. However, there is a potential for immune responses or the emergence of allergic reactions, which may be attributed to the presence of residual sericin proteins remaining in the fibroin following its processing.	Provides controllable mechanical conditions for the growth of granulation tissue and biointegration. Also contains pro‐regenerative Arg‐Gly‐Asp (RGD) tripeptide sequences.

Abbreviation: PLA, polylactid acid.

Natural materials demonstrated beneficial pro‐regeneration effects and absence of postoperative strictures or other complications, making them highly promising for the translation into clinical practice. Such materials have a rapid biodegradation rate and little or no inflammatory response. However, some studies using cell‐free collagen scaffolds reported complications in animals, which may be due to the length of the modeled urethral defect.^21^, ^26^ According to the outcomes of the studies we examined, SIS exhibits limited potential as a reconstructive material, as various investigations reported that experimental animals experienced complications and stricture formation. To overcome this limitation, researchers transfected fibroblasts with TIMP‐1 siRNA, but complete prevention of relapse was not achieved.^44^ BSM contains abundance of urinary tract specific ECM scaffolding that promote rapid urethral recellularization and high biocompatibility.^49^, ^50^ However, the lack of recent data after 2017 on urethral tissue engineering using BSM, in addition to the difficulty of its production, renders it impractical for medical use. The use of acellular carotid arteries for urethral tissue engineering is equally infeasible because it requires to obtain the cadaveric material.^24^ The integrity of cadaveric material can be influenced by the presence of atherosclerotic plaques or calcification, which can make it exceedingly fragile and unsuitable for employment in urethroplasty procedures. Due to the availability of commercial AM products,^61^ their suitability for use in tissue engineering has been studied. The process of decellularization leaves signaling molecules for epithelial regeneration and preserves basement membrane facilitating cellular adhesion.^62^ However, there was a lack of information on long‐term effects and prevention of complications. Altogether, the complexity of decellularization protocols and the risk of immune responses made decellularized matrices unpopular among surgeons. Our literature search revealed only one paper^63^ (Table S3 in Data S1) that reported using decellularized material in a clinical trial since 2015.

The number of research studies dedicated to cell‐seeded scaffolds has been increasing over the years. It is important to note that while various cell types have demonstrated favorable outcomes, the cell type most suited for clinical implementation would be one that is readily accessible. For instance, OK have been employed in numerous clinical trials and may prove to be a particularly promising option (Table S4 in Data S1). Oral mucosal tissue sampling is almost painless. Also, this cell type can maintain barrier functions similar to that of natural urothelium.

These cells can be cultivated and differentiated to form epithelial layer on the lumen surface of urethral scaffolds. However, there are considerations that the effectiveness of this cellular component can be limited by impacts of urine and surgical manipulations. A catheter inserted into the rabbit's urethra post‐surgery can adversely damage the surface of the scaffold due to the need to be frequently replaced to prevent infection. Moreover, rabbit urine contains phosphate crystals that can clog the catheter, necessitating regular changes. Consequently, the thin cellular layer within the urethral lumen is likely to sustain damage or be partially or entirely removed during catheter removal, thus inhibiting its regenerative capabilities. Therefore, it can be beneficial to distribute cells in the bulk of the scaffold and leave the reepithelialization to the host's tissue. In this context, the functionality of cells can be diversified; for instance, the addition of HUVECs, transfected fibroblasts, or hypoxia‐preconditioned ADSCs may be employed to enhance the vascularization of the scaffold^55^ or prevent fibrosis.^44^ Additionally, transfection of autologous progenitor cells with lentiviral vectors encoding the immunomodulatory peptide LL37 can be employed to prevent postoperative infection.^50^

We would like to point out that the composition of the scaffold material is likely the predominant factor influencing the success of postoperative outcomes. Utilization of natural polymers either alone or in combination with synthetic materials yields improved postoperative outcomes. Collagen remains the integral material for scaffold design due to its ability to promote organ‐specific regeneration. Cell‐free collagen matrices were shown to promote full clinical recovery including painless urination and sexual activity on such observation time points as 9 and 11 months.^38^ Several studies highlight complications with cell‐seeded scaffolds, indicating the irrelevance of these materials for future implementation into clinical practice. According to our analysis, scaffolds made of PLA,^53^ silk fibroin,^36^, ^54^ and unmodified SIS^44^, ^45^, ^46^ have led to adverse postoperative results in both cellular scaffolds and cell‐free matrices. The future of urethral tissue engineering lies in the compromise between immunogenicity and mechanical strength on one hand and a favorable rate of biodegradation and pro‐regeneration signaling on the other hand. The alternative strategy can be modification of natural polymer materials aimed to achieve pharmaceutical and genetic control over the biointegration and tissue response.

Most studies had a postoperative follow‐up period of less than 12 weeks, which made it difficult to assess regenerative capacity of the scaffolds. Only one article on the collagen tubular scaffold had a follow‐up period of 44 weeks.^38^ Data from studies with longer follow‐up periods is most valuable because complications from acellular matrices in patients typically develop after at least 6 months. Lin et al. reported that urethrocutaneous fistula was the most frequent complication and developed 9–15 months post‐surgery.^3^ It was reported that urethroplasty patients can begin to complain of difficulty urinating after 9 months.^4^ Further investigations into long‐term interactions between the artificial tissue and the host will shed light on both the impact of the scaffold materials and chronic inflammation on the outcomes of urethroplasty.

## 5 LIMITATIONS

Our review's findings should be interpreted acknowledging certain constraints. Initially, the preclinical trials encompassed in this review exhibit considerable heterogeneity. We undertook a comparative analysis of scaffolds of differing configurations (such as patch or tube), varying urethral defect sizes, and disparate postoperative follow‐up durations. Literature indicates that the repair of shorter urethral defects typically engenders fewer complications than those exceeding 2.5 cm. Consequently, we cannot assert the effectiveness of scaffolds that did not induce stricture formation on defects measuring 2–2.5 cm with absolute certainty. Moreover, we cannot affirm the efficacy of scaffolds with merely 1‐ or 2‐month follow‐up period, as only studies of a longer duration can convincingly validate the absence of fibrous tissue proliferation around the implant. Nonetheless, we endeavored to distill the maximum quantum of data from each study, inclusive of the rate of formation of epithelial and muscle layers. This information could potentially facilitate researchers in devising new hypotheses concerning the materials used for urethral repair under investigation.

Secondly, the outcome of urethroplasty could be influenced by a myriad of factors such as the expertise of the operating surgeon, variations in postoperative animal care, whether a single surgeon performed all procedures or multiple specialists were involved, among others. These elements were not always explicitly and comprehensively delineated in the articles. Therefore, we advocate for researchers to give due consideration to these aspects during the planning stage of their experiments, to comply with international guidelines concerning animal handling and to provide a more thorough description of animal handling procedures within their articles. A detailed account of this stage will assist in averting misinterpretations of postoperative outcomes.

Thirdly, only 12 out of 48 studies qualified for inclusion in the meta‐analysis, given that not all articles provided outcomes for the control groups. Consequently, we were unable to produce compelling evidence to endorse either cellular or acellular scaffolds. Nevertheless, we have succinctly encapsulated the data from all studies in tabular form, thereby enabling researchers to independently scrutinize papers concerning their materials or cell types of interest and subsequently formulate their own conclusions.

## 6 CONCLUSION

Based on the findings of our review, several conclusions can be drawn. Firstly, decellularized materials utilized for urethral reconstruction have become increasingly irrelevant due to their complex fabrication process and unsatisfactory outcomes in preclinical tests. Additionally, pure synthetic scaffolds without modifications and cellular components have resulted in complications and strictures in animal trials. Natural polymers were more widely utilized than synthetic materials to develop scaffolds. Secondly, the utilization of natural polymers either alone or in combination with synthetic materials yielded improved postoperative outcomes in animals than constructs solely composed of synthetic materials. Collagen was the most frequently employed natural material for scaffold construction. OK were the most available and efficacious cells in preclinical trials, having similar barrier functions to urothelium while being less traumatic to obtain through biopsy. The next conclusion that can be reached from our review is that acellular constructs are gaining, as a consequence of exhibiting postoperative outcomes that are comparable to cell‐seeded scaffolds. Tissue‐engineered constructs show satisfactory results with short follow‐up periods, although longer trials are necessary to translate the findings into clinical practice.

## AUTHOR CONTRIBUTIONS

**Natalia Chepelova:** Conceptualization; investigation; methodology; visualization; writing – original draft. **Guzel Sagitova:** Investigation; writing – original draft. **Daniel Munblit:** Methodology; writing – review and editing. **Aleksandr Suvorov:** Formal analysis; methodology; visualization. **Andrey Morozov:** Validation; writing – original draft. **Anastasia Shpichka:** Conceptualization; investigation. **Peter Glybochko:** Funding acquisition; project administration. **Peter Timashev:** Funding acquisition; project administration; supervision; writing – review and editing. **Denis Butnaru:** Conceptualization; funding acquisition; project administration; supervision; validation; writing – review and editing.

## FUNDING INFORMATION

The research was funded by the Russian Science Foundation grant no. 23‐15‐00481, https://rscf.ru/project/23-15-00481.

## CONFLICT OF INTEREST STATEMENT

The authors declare no conflicts of interest.

## Supporting information


**Data S1.** Supporting information.

## Data Availability

Data sharing is not applicable to this article as no new data were created or analyzed in this study.
