# Supplementary material for: The search for an optimal tissue‐engineered urethra model for clinical application based on preclinical trials in male animals: A systematic review and meta‐analysis
Source: Bioeng Transl Med. 2024 Jul 23;9(6):e10700. doi: 10.1002/btm2.10700 (PMC11558198; doi:10.1002/btm2.10700)
Supplement: Supplementary file 1 — Data S1. Supporting information. [file BTM2-9-e10700-s001.docx]

**Supporting Information**

**SUPPLEMENTAL METHODS**

Supplementary Table 1. Search Methodology

| **Database** | **Filters** | **Keywords** | **Final search string** | **Result** |
| --- | --- | --- | --- | --- |
| **PubMed** | Full text;  Other Animals; English;  2015 to 2022 | urethral stricture scaffold;  urethral scaffold;  tissue engineering urethrae | **-** | 24;  74;  111 |
| **Scopus** | **-** | **-** | TITLE-ABS-KEY ( ( "tissue engineer*" OR regenerat* OR fabricat* OR restorat* ) AND ( strictur* OR hypospad* ) AND ( matri* OR scaffold OR construct OR graft ) AND ( animal OR rabbit OR dog OR pig OR canine ) ) AND ( LIMIT-TO ( DOCTYPE , "ar" ) OR EXCLUDE ( DOCTYPE , "re" ) OR EXCLUDE ( DOCTYPE , "cp" ) OR EXCLUDE ( DOCTYPE , "ch" ) ) AND ( LIMIT-TO ( PUBYEAR , 2022 ) OR LIMIT-TO ( PUBYEAR , 2021 ) OR LIMIT-TO ( PUBYEAR , 2020 ) OR LIMIT-TO ( PUBYEAR , 2019 ) OR LIMIT-TO ( PUBYEAR , 2018 ) OR LIMIT-TO ( PUBYEAR , 2017 ) OR LIMIT-TO ( PUBYEAR , 2016 ) OR LIMIT-TO ( PUBYEAR , 2015 ) ) AND ( LIMIT-TO ( LANGUAGE , "English" ) ) | 55 |
| **PMC** | 2015/01/01 to 2022/12/31 | urethral AND stricture AND tissue AND engineering AND regenerative AND medicine AND scaffold AND stem AND cells | ("urethra"[MeSH Terms] OR "urethra"[All Fields] OR "urethral"[All Fields]) AND ("constriction, pathologic"[MeSH Terms] OR ("constriction"[All Fields] AND "pathologic"[All Fields]) OR "pathologic constriction"[All Fields] OR "stricture"[All Fields]) AND ("tissues"[MeSH Terms] OR "tissues"[All Fields] OR "tissue"[All Fields]) AND ("engineering"[MeSH Terms] OR "engineering"[All Fields]) AND regenerative[All Fields] AND ("medicine"[MeSH Terms] OR "medicine"[All Fields]) AND scaffold[All Fields] AND ("plant stems"[MeSH Terms] OR ("plant"[All Fields] AND "stems"[All Fields]) OR "plant stems"[All Fields] OR "stem"[All Fields] OR "microscopy, electron, scanning transmission"[MeSH Terms] OR ("microscopy"[All Fields] AND "electron"[All Fields] AND "scanning"[All Fields] AND "transmission"[All Fields]) OR "scanning transmission electron microscopy"[All Fields]) AND ("cells"[MeSH Terms] OR "cells"[All Fields]) AND ("2015/01/01"[PubDate] : "2022/12/31"[PubDate]). | 103 |

PubMed searched for three-word combinations. The filters "Full text, Humans, Other Animals, English" were applied for all phrases. Also, a temporary filter was installed from 2015 to 2022. For the word combination "urethral stricture scaffold" 22 results was found. By the combination of the words "urethral scaffold" 71 results were displayed. By the combination of the phrase "tissue engineering urethrae" 107 results were found.

In Scopus, the final search string looked like this: TITLE-ABS-KEY ( ( "tissue engineer*" OR regenerat* OR fabricat* OR restorat* ) AND ( strictur* OR hypospad* ) AND ( matri* OR scaffold OR construct OR graft ) AND ( animal OR rabbit OR dog OR pig OR canine ) ) AND ( LIMIT-TO ( DOCTYPE , "ar" ) OR EXCLUDE ( DOCTYPE , "re" ) OR EXCLUDE ( DOCTYPE , "cp" ) OR EXCLUDE ( DOCTYPE , "ch" ) ) AND ( LIMIT-TO ( PUBYEAR , 2022 ) OR LIMIT-TO ( PUBYEAR , 2021 ) OR LIMIT-TO ( PUBYEAR , 2020 ) OR LIMIT-TO ( PUBYEAR , 2019 ) OR LIMIT-TO ( PUBYEAR , 2018 ) OR LIMIT-TO ( PUBYEAR , 2017 ) OR LIMIT-TO ( PUBYEAR , 2016 ) OR LIMIT-TO ( PUBYEAR , 2015 ) ) AND ( LIMIT-TO ( LANGUAGE , "English" ) ). 50 results were found on this request.

In the PMC database, keyword searches looked like this: urethral AND stricture AND tissue AND engineering AND regenerative AND medicine AND scaffold AND stem AND cells. As a result of the search, 118 articles were found, of which 90 articles were published during the period 2015/01/01 to 2022/12/31. The final search string looked like this: ("urethra"[MeSH Terms] OR "urethra"[All Fields] OR "urethral"[All Fields]) AND ("constriction, pathologic"[MeSH Terms] OR ("constriction"[All Fields] AND "pathologic"[All Fields]) OR "pathologic constriction"[All Fields] OR "stricture"[All Fields]) AND ("tissues"[MeSH Terms] OR "tissues"[All Fields] OR "tissue"[All Fields]) AND ("engineering"[MeSH Terms] OR "engineering"[All Fields]) AND regenerative[All Fields] AND ("medicine"[MeSH Terms] OR "medicine"[All Fields]) AND scaffold[All Fields] AND ("plant stems"[MeSH Terms] OR ("plant"[All Fields] AND "stems"[All Fields]) OR "plant stems"[All Fields] OR "stem"[All Fields] OR "microscopy, electron, scanning transmission"[MeSH Terms] OR ("microscopy"[All Fields] AND "electron"[All Fields] AND "scanning"[All Fields] AND "transmission"[All Fields]) OR "scanning transmission electron microscopy"[All Fields]) AND ("cells"[MeSH Terms] OR "cells"[All Fields]) AND ("2015/01/01"[PubDate] : "2022/12/31"[PubDate]).

Supplementary Table 2. Article selection

|  | **Title of the article (Screening; n= 229)** | **Full-text article assesed for eligability (Eligability; n=67)** | **Meets inclusion criteria (Included; n=48)** |
| --- | --- | --- | --- |
| 1 | The application of dried amniotic membrane s+A2:A3caffold with adipose derived-mesenchymal stem cell seeding as graft in urethral reconstruction (experiment on rabbit) | Yes | Yes |
| 2 | Biomaterial-Based Scaffolds as Antibacterial Suture Materials | Yes | Yes |
| 3 | Urethral tissue regeneration using collagen scaffold modified with collagen binding VEGF in a beagle model | Yes | Yes |
| 4 | Urethral Reconstruction Using Mesothelial Cell-Seeded Autogenous Granulation Tissue Tube: An Experimental Study in Male Rabbits. | Yes | Yes |
| 5 | Prevascularized bladder acellular matrix hydrogel/silk fibroin composite scaffolds promote the regeneration of urethra in a rabbit model. | Yes | Yes |
| 6 | Electrospun PLGA and PLGA/gelatin scaffolds for tubularized urethral replacement: Studies in vitro and in vivo | Yes | Yes |
| 7 | Stretchable collagen-coated polyurethane-urea hydrogel seeded with bladder smooth muscle cells for urethral defect repair in a rabbit model | Yes | Yes |
| 8 | Structural and functional evaluation of oxygenating keratin/silk fibroin scaffold and initial assessment of their potential for urethral tissue engineering | Yes | Yes |
| 9 | Electrospun nanoyarn and exosomes of adipose-derived stem cells for urethral regeneration: Evaluations in vitro and in vivo | Yes | Yes |
| 10 | Fabrication of tissue-engineered bionic urethra using cell sheet technology and labeling by ultrasmall superparamagnetic iron oxide for full-thickness urethral reconstruction | Yes | Yes |
| 11 | Urethra-inspired biomimetic scaffold: A therapeutic strategy to promote angiogenesis for urethral regeneration in a rabbit model. | Yes | Yes |
| 12 | Urethroplasty using autologous urethral tissue-embedded acellular porcine bladder submucosa matrix grafts for the management of long-segment urethral stricture in a rabbit model | Yes | Yes |
| 13 | Sustained release of stromal cell-derived factor-1 alpha from silk fibroin microfiber promotes urethral reconstruction in rabbits. | Yes | Yes |
| 14 | Reconstruction of rabbit urethral epithelium with skin keratinocytes | Yes | Yes |
| 15 | A Preclinical Study of Cell-seeded Tubularized Scaffolds Specially Secreting LL37 for Reconstruction of Long Urethral Defects | Yes | Yes |
| 16 | Urethral Reconstruction with Small Intestinal Submucosa Seeded with Oral Keratinocytes and TIMP-1 siRNA Transfected Fibroblasts in a Rabbit Model | Yes | Yes |
| 17 | Urethral reconstruction with autologous urine-derived stem cells seeded in three-dimensional porous small intestinal submucosa in a rabbit model | Yes | Yes |
| 18 | Effective Reconstruction of Functional Urethra Promoted With ICG-001 Delivery Using Core-Shell Collagen/Poly(Llactide-co-caprolactone) [P(LLA-CL)] Nanoyarn-Based Scaffold: A Study in Dog Model | Yes | Yes |
| 19 | Application of Wnt pathway inhibitor delivering scaffold for inhibiting fibrosis in urethra strictures: In vitro and in vivo study | Yes | Yes |
| 20 | Cryopreserved skin epithelial cell sheet combined with acellular amniotic membrane as an off-the-shelf scaffold for urethral regeneration. | Yes | Yes |
| 21 | Urethral reconstruction using an amphiphilic tissue-engineered autologous polyurethane nanofiber scaffold with rapid vascularization function. | Yes | Yes |
| 22 | Anterior substitutional urethroplasty using a biomimetic poly-l-lactide nanofiber membrane: Preclinical and clinical outcomes | Yes | Yes |
| 23 | Repair of urethral defects with polylactid acid fibrous membrane seeded with adipose-derived stem cells in a rabbit model. | Yes | Yes |
| 24 | Urethroplasty performed with an autologous urothelium-vegetated collagen fleece to treat urethral stricture in the minipig model | Yes | Yes |
| 25 | Collagen cell carriers seeded with human urothelial cells for urethral reconstructive surgery: first results in a xenograft minipig model | Yes | Yes |
| 26 | Electrospun poly(L-lactide)/poly(ethylene glycol) scaffolds seeded with human amniotic mesenchymal stem cells for urethral epithelium repair | Yes | Yes |
| 27 | Development of a cell-seeded modified small intestinal submucosa for urethroplasty | Yes | Yes |
| 28 | Cell-Seeded Acellular Artery for Reconstruction of Long Urethral Defects in a Canine Model. | Yes | Yes |
| 29 | Double-Modified Bacterial Cellulose/Soy Protein Isolate Composites by Laser Hole Forming and Selective Oxidation Used for Urethral Repair. | Yes | Yes |
| 30 | Creation of Tissue-Engineered Urethras for Large Urethral Defect Repair in a Rabbit Experimental Model. | Yes | Yes |
| 31 | Urethroplasty with a bilayered poly-D,L-lactide-co-ε-caprolactone scaffold seeded with allogenic mesenchymal stem cells. | Yes | Yes |
| 32 | HA-coated collagen nanofibers for urethral regeneration via in situ polarization of M2 macrophages | Yes | Yes |
| 33 | Tissue-engineered PLLA/gelatine nanofibrous scaffold promoting the phenotypic expression of epithelial and smooth muscle cells for urethral reconstruction. | Yes | Yes |
| 34 | Comparison of Poly(l-lactide-co-ϵ-caprolactone) and Poly(trimethylene carbonate) Membranes for Urethral Regeneration: An in Vitro and in Vivo Study | Yes | Yes |
| 35 | Application of Tissue Engineering Construct Seeded with Buccal Epithelium Cells for Replacement Urethroplasty | Yes | Yes |
| 36 | Repair of injured urethras with silk fibroin scaffolds in a rabbit model of onlay urethroplasty. | Yes | Yes |
| 37 | Engineered acellular collagen scaffold for endogenous cell guidance, a novel approach in urethral regeneration. | Yes | Yes |
| 38 | Urethral reconstruction with a 3D porous bacterial cellulose scaffold seeded with lingual keratinocytes in a rabbit model | Yes | Yes |
| 39 | An extracellular matrix-mimicking, bilayered, heterogeneous, porous, nanofibrous scaffold for anterior urethroplasty in a rabbit model. | Yes | Yes |
| 40 | Designing a multifaceted bio-interface nanofiber tissue-engineered tubular scaffold graft to promote neo-vascularization for urethral regeneration. | Yes | Yes |
| 41 | Transplantation of Amniotic Scaffold-Seeded Mesenchymal Stem Cells and/or Endothelial Progenitor Cells From Bone Marrow to Efficiently Repair 3-cm Circumferential Urethral Defect in Model Dogs. | Yes | Yes |
| 42 | Fiber density of collagen grafts impacts rabbit urethral regeneration. | Yes | Yes |
| 43 | Repair of urethral defects by an adipose mesenchymal stem cell‑porous silk fibroin material | Yes | Yes |
| 44 | Hypoxia-preconditioned adipose-derived stem cells combined with scaffold promote urethral reconstruction by upregulation of angiogenesis and glycolysis. | Yes | Yes |
| 45 | Collagen scaffolds tethered with bFGF promote corpus spongiosum regeneration in a beagle model. | Yes | Yes |
| 46 | Urethral Tissue Reconstruction Using the Acellular Dermal Matrix Patch Modified with Collagen-Binding VEGF in Beagle Urethral Injury Models. | Yes | Yes |
| 47 | A biomimetic hyaluronic acid-silk fibroin nanofiber scaffold promoting regeneration of transected urothelium | Yes | Yes |
| 48 | Clinical application of a double-modified sulfated bacterial cellulose scaffold material loaded with FGFR2-modified adipose-derived stem cells in urethral reconstruction | Yes | Yes |
| 49 | Tissue-engineered tubular substitutions for urinary diversion in a rabbit model | Yes | No (inappropriate topic) |
| 50 | A smart bilayered scaffold supporting keratinocytes and muscle cells in micro/nano-scale for urethral reconstruction. | Yes | No (female animal model) |
| 51 | Penile urethra replacement with autologous cell-seeded tubularized collagen matrices. | Yes | No (inappropriate year of publication - 2012) |
| 52 | Off-the-shelf acellular fetal skin scaffold as a novel alternative to buccal mucosa graft: the development and characterization of human tissue-engineered fetal matrix in rabbit model of hypospadiasis | Yes | No (inappropriate topic) |
| 53 | Buccal epithelium Expanded and Encapsulated in Scaffold-Hybrid Approach to Urethral Stricture (BEES-HAUS) procedure: A novel cell therapy-based pilot study. | Yes | No (a clinical trial) |
| 54 | Developing improved tissue-engineered buccal mucosa grafts for urethral reconstruction. | Yes | No (no animal model) |
| 55 | Histological and morphometric evaluation of the urethra and penis in male New Zealand White rabbits. | Yes | No (inappropriate topic) |
| 56 | New Amniotic Membrane Based Biocomposite for Future Application in Reconstructive Urology. | Yes | No (rat animal model) |
| 57 | Clinical challenges in tissue-engineered urethral reconstruction | Yes | No (a review) |
| 58 | Reconstructive urology and tissue engineering: Converging developmental paths. | Yes | No (a review) |
| 59 | Growth factor and small molecule influence on urological tissue regeneration utilizing cell seeded scaffolds. | Yes | No (inappropriate topic) |
| 60 | Autologous granulation tissue tubes for replacement of urethral defects: An experimental study in male rabbits | Yes | No (inappropriate topic) |
| 61 | Mechanical induction of bi-directional orientation of primary porcine bladder smooth muscle cells in tubular fibrin-poly(vinylidene fluoride) scaffolds for ureteral and urethral repair using cyclic and focal balloon catheter stimulation | Yes | No (rat animal model) |
| 62 | Dynamically crosslinked polydimethylsiloxane-based polyurethanes with contact-killing antimicrobial properties as implantable alloplasts for urological reconstruction. | Yes | No (no animal model) |
| 63 | Biocompatibility of different nanostructured TiO(2) scaffolds and their potential for urologic applications. | Yes | No (no animal model) |
| 64 | Tissue engineered pre-vascularized buccal mucosa equivalents utilizing a primary triculture of epithelial cells, endothelial cells and fibroblasts. | Yes | No (no animal model) |
| 65 | Fabrication of Adipose-Derived Stem Cell-Based Self-Assembled Scaffold under Hypoxia and Mechanical Stimulation for Urethral Tissue Engineering | Yes | No (no animal model) |
| 66 | Effects of Anti-Inflammatory Nanofibers on Urethral Healing. | Yes | No (rat animal model) |
| 67 | Future Prospects for Human Tissue Engineered Urethra Transplantation: Decellularization and Recellularization-Based Urethra Regeneration. | Yes | No (rat animal model) |
| 68 | Plumping up a Cushion of Human Biowaste in Regenerative Medicine: Novel Insights into a State-of-the-Art Reserve Arsenal | No (inappropriate topic ) |  |
| 69 | The Growing Medical Need for Tracheal Replacement: Reconstructive Strategies Should Overcome Their Limits | No (inappropriate topic ) |  |
| 70 | ADSC-sheet transplantation to prevent stricture after extended esophageal endoscopic submucosal dissection | No (inappropriate topic ) |  |
| 71 | Molecular Cancer Imaging in the Second Near-Infrared Window Using a Renal-Excreted NIR-II Fluorophore-Peptide Probe. | No (inappropriate topic ) |  |
| 72 | Cryopreservation of porcine urethral tissue: Storage at -20°C preserves the mechanical, failure and geometrical properties. | No (inappropriate topic ) |  |
| 73 | Preconditioning and Engineering Strategies for Improving the Efficacy of Mesenchymal Stem Cell-Derived Exosomes in Cell-Free Therapy | No (inappropriate topic ) |  |
| 74 | Arterial Thromboembolism | No (inappropriate topic ) |  |
| 75 | Successful muscle regeneration by a homologous microperforated scaffold seeded with autologous mesenchymal stromal cells in a porcine esophageal substitution model | No (inappropriate topic ) |  |
| 76 | A review on biodegradable biliary stents: materials and future trends | No (inappropriate topic ) |  |
| 77 | Therapeutic effect of dental pulp stem cell transplantation on a rat model of radioactivity-induced esophageal injury | No (inappropriate topic ) |  |
| 78 | Evaluation of Selected Properties of Sodium Alginate-Based Hydrogel Material-Mechanical Strength, μDIC Analysis and Degradation | No (inappropriate topic ) |  |
| 79 | Real-time temperature monitoring with fiber Bragg grating sensor during diffuser-assisted laser-induced interstitial thermotherapy | No (inappropriate topic ) |  |
| 80 | A dynamic distention protocol for whole-organ bladder decellularization: histological and biomechanical characterization of the acellular matrix. | No (inappropriate topic ) |  |
| 81 | Two cases of female urethral reconstruction with acellular porcine urinary bladder matrix. | No (inappropriate topic ) |  |
| 82 | Reversal of senescence-associated beta-galactosidase expression during in vitro three-dimensional tissue-engineering of human chondrocytes in a polymer scaffold | No (inappropriate topic ) |  |
| 83 | Targeted therapy for stress urinary incontinence: a systematic review based on clinical trials. | No (inappropriate topic ) |  |
| 84 | Bi-layer silk fibroin grafts support functional tissue regeneration in a porcine model of onlay esophagoplasty | No (inappropriate topic ) |  |
| 85 | Bladder reconstruction using autologous smooth muscle cell sheets grafted on a pre-vascularized capsule | No (inappropriate topic ) |  |
| 86 | Evaluation of Bilayer Silk Fibroin Grafts for Tubular Esophagoplasty in a Porcine Defect Model | No (inappropriate topic ) |  |
| 87 | Local release from affinity-based polymers increases urethral concentration of the stem cell chemokine CCL7 in rats. | No (inappropriate topic ) |  |
| 88 | Biocompatible Polymer Materials with Antimicrobial Properties for Preparation of Stents | No (inappropriate topic ) |  |
| 89 | Multiple doses of stem cells maintain urethral function in a model of neuromuscular injury resulting in stress urinary incontinence. | No (inappropriate topic ) |  |
| 90 | Modeling Esophagitis Using Human Three-Dimensional Organotypic Culture System | No (inappropriate topic ) |  |
| 91 | Bioscaffold-mediated mucosal remodeling following short-segment colonic mucosal resection | No (inappropriate topic ) |  |
| 92 | International Union of Basic and Clinical Pharmacology. CX. Classification of Receptors for 5-hydroxytryptamine; Pharmacology and Function | No (inappropriate topic ) |  |
| 93 | Applicability of regenerative medicine and tissue engineering for the treatment of stress urinary incontinence in female patients. | No (inappropriate topic ) |  |
| 94 | Tissue-specific roles of FGF signaling in external genitalia development. | No (inappropriate topic ) |  |
| 95 | WNT Signaling in Cardiac and Vascular Disease | No (inappropriate topic ) |  |
| 96 | An Instrumented Urethral Catheter with a Distributed Array of Iontronic Force Sensors. | No (inappropriate topic ) |  |
| 97 | Advancing biomaterials of human origin for tissue engineering | No (inappropriate topic ) |  |
| 98 | Applications of regenerative medicine in organ transplantation | No (inappropriate topic ) |  |
| 99 | Design, Synthesis, and Biological Evaluation of Novel Tetrahydroprotoberberine Derivatives (THPBs) as Selective α(1A)-Adrenoceptor Antagonists. | No (inappropriate topic ) |  |
| 100 | Gel Casting as an Approach for Tissue Engineering of Multilayered Tubular Structures | No (inappropriate topic ) |  |
| 101 | An artificial bile duct made of bioabsorbable polymer: A viable substitute for narrowed portion of the extrahepatic bile duct | No (inappropriate topic ) |  |
| 102 | Progress of esophageal stricture prevention after endoscopic submucosal dissection by regenerative medicine and tissue engineering | No (inappropriate topic ) |  |
| 103 | Experimental investigation of the biomechanics of urethral tissues and structures. | No (inappropriate topic ) |  |
| 104 | Application of Dried Human Amnion Graft to Improve Post-Prostatectomy Incontinence and Potency: A Randomized Exploration Study Protocol | No (inappropriate topic ) |  |
| 105 | Reconstruction of the mouse extrahepatic biliary tree using primary human extrahepatic cholangiocyte organoids | No (inappropriate topic ) |  |
| 106 | EW-7197 eluting nano-fiber covered self-expandable metallic stent to prevent granulation tissue formation in a canine urethral model. | No (inappropriate topic ) |  |
| 107 | Histological and Biochemical Evaluation of Urethral Scar following Three Different Hypospadias Repairs: An Experimental Study in Rabbits. | No (inappropriate topic ) |  |
| 108 | In vivo human corpus cavernosum regeneration: fabrication of tissue-engineered corpus cavernosum in rat using the body as a natural bioreactor. | No (inappropriate topic ) |  |
| 109 | Urine as a Main Effector in Urological Tissue Engineering-A Double-Edged Sword | No (inappropriate topic ) |  |
| 110 | Systematic Review to Compare Urothelium Differentiation with Urethral Epithelium Differentiation in Fetal Development, as a Basis for Tissue Engineering of the Male Urethra | No (inappropriate topic ) |  |
| 111 | Effects of Thermal Preconditioning on Tissue Susceptibility to Histotripsy. | No (inappropriate topic ) |  |
| 112 | Comparative Assessment of Cultures from Oral and Urethral Stem Cells for Urethral Regeneration. | No (inappropriate topic ) |  |
| 113 | Current Knowledge and Future Perspectives on Mesenchymal Stem Cell-Derived Exosomes as a New Therapeutic Agent | No (inappropriate topic ) |  |
| 114 | Mechanics of the urethral duct: tissue constitutive formulation and structural modeling for the investigation of lumen occlusion. | No (inappropriate topic ) |  |
| 115 | Electrospinning: Application and Prospects for Urologic Tissue Engineering | No (inappropriate topic ) |  |
| 116 | Acellular bi-layer silk fibroin scaffolds support functional tissue regeneration in a rat model of onlay esophagoplasty | No (inappropriate topic ) |  |
| 117 | 3D bioprinting of urethra with PCL/PLCL blend and dual autologous cells in fibrin hydrogel: An in vitro evaluation of biomimetic mechanical property and cell growth environment | No (inappropriate topic ) |  |
| 118 | Repair of extrahepatic bile duct defect using a collagen patch in a swine model | No (inappropriate topic ) |  |
| 119 | A Novel Method of Urinary Sphincter Deficiency: Serial Histopathology Evaluation in a Rat Model of Urinary Incontinence. | No (inappropriate topic ) |  |
| 120 | Smooth Muscle Progenitor Cells Derived From Human Pluripotent Stem Cells Induce Histologic Changes in Injured Urethral Sphincter. | No (inappropriate topic ) |  |
| 121 | Does pharmacological activation of 5-HT1A receptors improve urine flow rate in female rats? | No (inappropriate topic ) |  |
| 122 | Production of bacterial cellulose tubes for biomedical applications: Analysis of the effect of fermentation time on selected properties. | No (inappropriate topic ) |  |
| 123 | Tubular organ epithelialisation | No (inappropriate topic ) |  |
| 124 | In vitro culture of rat hair follicle stem cells on rabbit bladder acellular matrix | No (inappropriate topic ) |  |
| 125 | TGF-β1 relieves epithelial-mesenchymal transition reduction in hypospadias induced by DEHP in rats. | No (inappropriate topic ) |  |
| 126 | Onlay foreskin flap anastomosed directly to the tunica albuginea: A short-term experimental study in rabbits | No (inappropriate topic ) |  |
| 127 | Elastic large muscular vessel wall engineered with bone marrow‑derived cells under pulsatile stimulation in a bioreactor. | No (inappropriate topic ) |  |
| 128 | Preclinical Evaluation of the Safety and Immunological Action of Allogeneic ADSC-Collagen Scaffolds in the Treatment of Chronic Ischemic Cardiomyopathy | No (inappropriate topic ) |  |
| 129 | Evaluation of Bi-Layer Silk Fibroin Grafts for Tubular Ureteroplasty in a Porcine Defect Model | No (inappropriate topic ) |  |
| 130 | Extracellular vesicles derived from different sources of mesenchymal stem cells: therapeutic effects and translational potential | No (inappropriate topic ) |  |
| 131 | Effects of pudendal neuromodulation on bladder function in chronic spinal cord-injured rats. | No (inappropriate topic ) |  |
| 132 | Preclinical study for treatment of hypospadias by advanced therapy medicinal products | No (inappropriate topic ) |  |
| 133 | Mesenchymal stem cell therapy in a rat model of birth-trauma injury: functional improvements and biodistribution. | No (inappropriate topic ) |  |
| 134 | Bladder reconstruction with human amniotic membrane in a xenograft rat model: A preclinical study | No (inappropriate topic ) |  |
| 135 | Testosterone and Estrogen Repletion in a Hypogonadal Environment Improves Post-operative Angiogenesis. | No (inappropriate topic ) |  |
| 136 | Novel Techniques to Improve Precise Cell Injection. | No (inappropriate topic ) |  |
| 137 | The potential role of regenerative medicine in the man-agement of traumatic patients | No (inappropriate topic ) |  |
| 138 | Balloon-Expandable Biodegradable Stents Versus Self-Expandable Metallic Stents: A Comparison Study of Stent-Induced Tissue Hyperplasia in the Rat Urethra. | No (inappropriate topic ) |  |
| 139 | Scaffolds for whole organ tissue engineering: Construction and in vitro evaluation of a seamless, spherical and hollow collagen bladder construct with appendices. | No (inappropriate topic ) |  |
| 140 | Healing effects of a protein scaffold loaded with adipose-derived mesenchymal stem cells on radiation-induced vaginal injury in rats | No (inappropriate topic ) |  |
| 141 | In vivo molecular engineering of the urethra for treatment of stress incontinence using novel biomimetic proteoglycans. | No (inappropriate topic ) |  |
| 142 | Tissue Engineering and Its Potential to Reduce Prostate Cancer Treatment Sequelae-Narrative Review | No (inappropriate topic ) |  |
| 143 | Vaginal wall weakness in parous ewes: a potential preclinical model of pelvic organ prolapse. | No (inappropriate topic ) |  |
| 144 | Exploratory safety study of an umbilical cord derived urethral sling in bilateral pudendal nerves injury-induced urinary incontinence in female rats. | No (inappropriate topic ) |  |
| 145 | Controlled release of insulin-like growth factor 1 enhances urethral sphincter function and histological structure in the treatment of female stress urinary incontinence in a rat model. | No (inappropriate topic ) |  |
| 146 | Effect of Pregnancy and Delivery on Cytokine Expression in a Mouse Model of Pelvic Organ Prolapse. | No (inappropriate topic ) |  |
| 147 | Human biliary epithelial cells from discarded donor livers rescue bile duct structure and function in a mouse model of biliary disease | No (inappropriate topic ) |  |
| 148 | A tubular gelatin scaffold capable of the time-dependent controlled release of epidermal growth factor and mitomycin C. | No (inappropriate topic ) |  |
| 149 | The fabrication of 3D surface scaffold of collagen/poly (L-lactide-co-caprolactone) with dynamic liquid system and its application in urinary incontinence treatment as a tissue engineered sub-urethral sling: In vitro and in vivo study. | No (inappropriate topic ) |  |
| 150 | Analysis of the Physico-Chemical, Mechanical and Biological Properties of Crosslinked Type-I Collagen from Horse Tendon: Towards the Development of Ideal Scaffolding Material for Urethral Regeneration | No (inappropriate topic ) |  |
| 151 | Decellularized dermal strip as a suburethral sling in a rat model of stress urinary incontinence. | No (inappropriate topic ) |  |
| 152 | Effect of adipose tissue-derived stem cell injection in a rat model of urethral fibrosis | No (inappropriate topic ) |  |
| 153 | Age is associated with reduced urethral pressure and afferent activity in rat. | No (inappropriate topic ) |  |
| 154 | Adhesion of fibroblast cells on thin films representing surfaces of polymeric scaffolds of human urethra rationalized by molecular models of integrin binding: cell adhesion on polymeric scaffolds for regenerative medicine. | No (inappropriate topic ) |  |
| 155 | Experimental study on the repair of ureteral functional regeneration with highly bioactive extracellular matrix stent | No (inappropriate topic ) |  |
| 156 | Urothelium with barrier function differentiated from human urine-derived stem cells for potential use in urinary tract reconstruction. | No (inappropriate topic ) |  |
| 157 | Clinical applications of acellular dermal matrices: A review | No (inappropriate topic ) |  |
| 158 | Use of bioactive extracellular matrix fragments as a urethral bulking agent to treat stress urinary incontinence. | No (inappropriate topic ) |  |
| 159 | Contribution of amniotic membrane to the healing of iatrogenic vas deferens injury | No (inappropriate topic ) |  |
| 160 | Acellular Urethra Bioscaffold: Decellularization of Whole Urethras for Tissue Engineering Applications. | No (inappropriate topic ) |  |
| 161 | Bioengineering Approaches for Bladder Regeneration | No (inappropriate topic ) |  |
| 162 | Modulatory effects of intravesical P2X2/3 purinergic receptor inhibition on lower urinary tract electromyographic properties and voiding function of female rats with moderate or severe spinal cord injury. | No (inappropriate topic ) |  |
| 163 | Sirolimus-eluting Biodegradable Poly-l-Lactic Acid Stent to Suppress Granulation Tissue Formation in the Rat Urethra. | No (inappropriate topic ) |  |
| 164 | Therapeutic candidates for keloid scars identified by qualitative review of scratch assay research for wound healing | No (inappropriate topic ) |  |
| 165 | Discovery of novel pyrazolopyrimidinone analogs as potent inhibitors of phosphodiesterase type-5. | No (inappropriate topic ) |  |
| 166 | Bile duct reconstruction using scaffold-free tubular constructs created by Bio-3D printer | No (inappropriate topic ) |  |
| 167 | Characterization of rabbit urine-derived stem cells for potential application in lower urinary tract tissue regeneration. | No (inappropriate topic ) |  |
| 168 | Retardation of Preputial Wound Healing in Rats with Hypospadias Induced by Flutamide. | No (inappropriate topic ) |  |
| 169 | Viability and MR detectability of iron labeled mesenchymal stem cells used for endoscopic injection into the porcine urethral sphincter. | No (inappropriate topic ) |  |
| 170 | Bone Marrow Stem/Progenitor Cells Attenuate the Inflammatory Milieu Following Substitution Urethroplasty | No (inappropriate topic ) |  |
| 171 | Transplantation of oral mucosal epithelial cells seeded on decellularized and lyophilized amniotic membrane for the regeneration of injured endometrium | No (inappropriate topic ) |  |
| 172 | A composite scaffold fabricated with an acellular matrix and biodegradable polyurethane for the in vivo regeneration of pig bile duct defects. | No (inappropriate topic ) |  |
| 173 | Postoperative imaging of living donor liver transplantation complications | No (inappropriate topic ) |  |
| 174 | Considerations for the clinical use of stem cells in genitourinary regenerative medicine | No (inappropriate topic ) |  |
| 175 | Abstracts of Scientific Papers 2017 AALAS National Meeting | No (conference materials) |  |
| 176 | The 45th Annual Meeting of the European Society for Blood and Marrow Transplantation: Physicians - Poster Session | No (conference materials) |  |
| 177 | UEG Week 2019 Poster Presentations | No (conference materials) |  |
| 178 | UEG Week 2018 Poster Presentations | No (conference materials) |  |
| 179 | 2016 ACVIM Forum Research Report Program | No (conference materials) |  |
| 180 | Abstracts : 31 (st) European Congress of Pathology | No (conference materials) |  |
| 181 | BNA 2021 Festival of Neuroscience Poster abstracts | No (conference materials) |  |
| 182 | UEG Week 2017 Poster Presentations | No (conference materials) |  |
| 183 | Scientific Session of the 16th World Congress of Endoscopic Surgery, Jointly Hosted by Society of American Gastrointestinal and Endoscopic Surgeons (SAGES) & Canadian Association of General Surgeons (CAGS), Seattle, Washington, USA, 11-14 April 2018: Post | No (conference materials) |  |
| 184 | Urethral reconstruction with RNA interference and polycaprolactone/silk fibroin/collagen electrospun fiber in rabbits | No (full-text version is not available) |  |
| 185 | Human Amniotic Membrane and Amniotic Membrane-Derived Cells: How Far Are We from Their Use in Regenerative and Reconstructive Urology? | No (a review) |  |
| 186 | Recent Advances in treatment of urethral stricture disease in men | No (a review) |  |
| 187 | Onlay Repair Technique for the Management of Ureteral Strictures: A Comprehensive Review | No (a review) |  |
| 188 | Frontiers in urethra regeneration: current state and future perspective. | No (a review) |  |
| 189 | A brief review on anterior urethral strictures | No (a review) |  |
| 190 | Genitourinary Tissue Engineering: Reconstruction and Research Models | No (a review) |  |
| 191 | Urothelial or oral mucosa cells for tissue-engineered urethroplasty: A critical revision of the clinical outcome | No (a review) |  |
| 192 | Naturally-Derived Biomaterials for Tissue Engineering Applications | No (a review) |  |
| 193 | Application of amniotic membrane in reconstructive urology; the promising biomaterial worth further investigation. | No (a review) |  |
| 194 | Current state of urethral tissue engineering. | No (a review) |  |
| 195 | Regenerative and engineered options for urethroplasty. | No (a review) |  |
| 196 | Tissue Engineering and Stem Cell Therapy in Pediatric Urology | No (a review) |  |
| 197 | Tissue engineered buccal mucosa for urethroplasty: progress and future directions | No (a review) |  |
| 198 | Tissue engineering: recent advances and review of clinical outcome for urethral strictures. | No (a review) |  |
| 199 | Cultured epidermal stem cells in regenerative medicine. | No (a review) |  |
| 200 | The potential role of tissue-engineered urethral substitution: clinical and preclinical studies. | No (a review) |  |
| 201 | A Scarless Healing Tale: Comparing Homeostasis and Wound Healing of Oral Mucosa With Skin and Oesophagus | No (a review) |  |
| 202 | Tissue engineering for urinary tract reconstruction and repair: Progress and prospect in China | No (a review) |  |
| 203 | Current Status of Tissue Engineering in the Management of Severe Hypospadias | No (a review) |  |
| 204 | Expression of Mafb is down-regulated in the foreskin of children with hypospadias. | No (a review) |  |
| 205 | The Fabrication and Evaluation of a Potential Biomaterial Produced with Stem Cell Sheet Technology for Future Regenerative Medicine | No (a review) |  |
| 206 | The current state of tissue engineering in the management of hypospadias. | No (a review) |  |
| 207 | Bioengineered Scaffolds as Substitutes for Grafts for Urethra Reconstruction | No (a review) |  |
| 208 | Stem cell in urology-are we at the cusp of a new era? | No (a review) |  |
| 209 | Tissue engineering in pediatric urology - a critical appraisal | No (a review) |  |
| 210 | Amniotic therapeutic biomaterials in urology: current and future applications | No (a review) |  |
| 211 | Silk Fibroin Scaffolds for Urologic Tissue Engineering | No (a review) |  |
| 212 | Biofabrication and biomaterials for urinary tract reconstruction | No (a review) |  |
| 213 | From Acellular Matrices to Smart Polymers: Degradable Scaffolds that are Transforming the Shape of Urethral Tissue Engineering | No (a review) |  |
| 214 | A state-of-the-art review of the fabrication and characteristics of titanium and its alloys for biomedical applications | No (a review) |  |
| 215 | Tissue Engineering and Regenerative Medicine: Achievements, Future, and Sustainability in Asia | No (a review) |  |
| 216 | Concise Review: Tissue Engineering of Urinary Bladder; We Still Have a Long Way to Go? | No (a review) |  |
| 217 | Nanogels for regenerative medicine | No (a review) |  |
| 218 | Overview of Urethral Reconstruction by Tissue Engineering: Current Strategies, Clinical Status and Future Direction | No (a review) |  |
| 219 | Acellular matrix in urethral reconstruction. | No (a review) |  |
| 220 | Cells Involved in Urethral Tissue Engineering: Systematic Review | No (a systematic review) |  |
| 221 | A systematic review on cell-seeded tissue engineering of penile corpora. | No (a systematic review) |  |
| 222 | Tissue Engineering of the Urethra: A Systematic Review and Meta-analysis of Preclinical and Clinical Studies | No (a systematic review) |  |
| 223 | Tissue Engineering of the Urethra: From Bench to Bedside | No (a systematic review) |  |
| 224 | Seeding cell approach for tissue-engineered urethral reconstruction in animal study: A systematic review and meta-analysis | No (a systematic review) |  |
| 225 | A systematic review of animal and clinical studies on the use of scaffolds for urethral repair. | No (a systematic review) |  |
| 226 | Tissue engineering of urethra: Systematic review of recent literature. | No (a systematic review) |  |
| 227 | Tissue engineering for human urethral reconstruction: systematic review of recent literature. | No (a systematic review) |  |
| 228 | Quality of Reporting in Preclinical Urethral Tissue Engineering Studies: A Systematic Review to Assess Adherence to the ARRIVE Guidelines | No (a systematic review) |  |
| 229 | Tissue-Engineered Grafts from Human Decellularized Extracellular Matrices: A Systematic Review and Future Perspectives | No (a systematic review) |  |

**SUPPLEMENTAL FIGURES**

## Complications

##
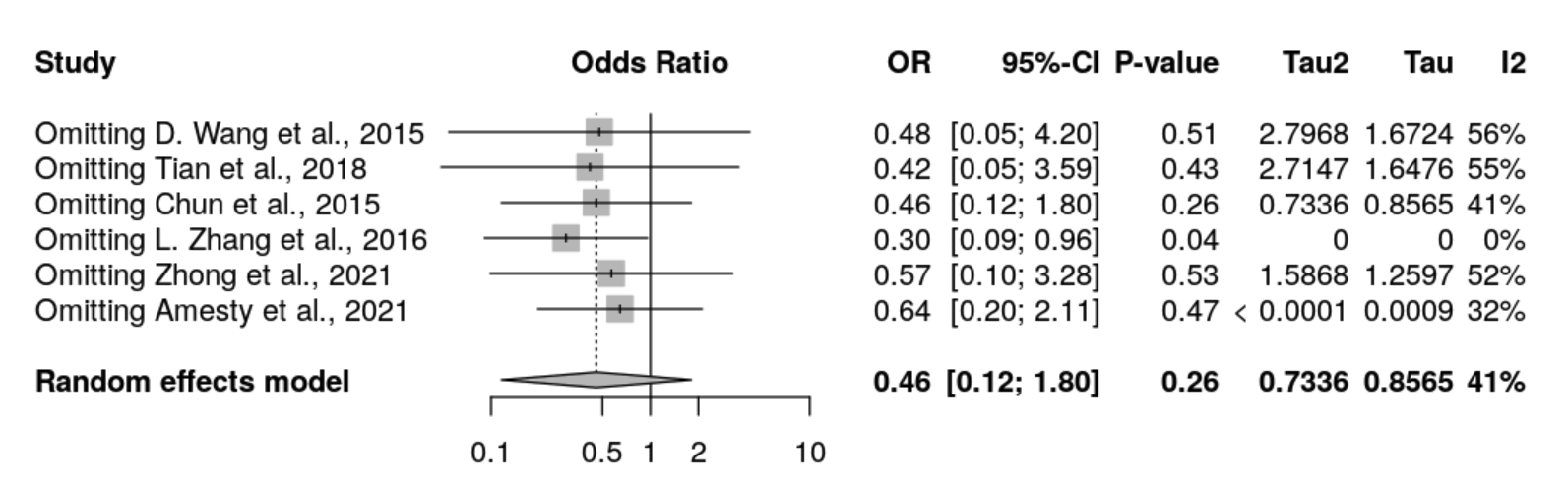


Figure S1. Sensitivity analysis (leave-one-out approach) for complications endpoint

Peters’ test results

Test result: t = 0.70, df = 3, p-value = 0.5321

Sample estimates:

bias se.bias intercept se.intercept

36.1970 51.4040 -2.8446 2.8656

Details:

- multiplicative residual heterogeneity variance (tau^2 = 13.3937)

- predictor: inverse of total sample size

- weight: inverse variance of average event probability

- reference: Peters et al. (2006), JAMA

##
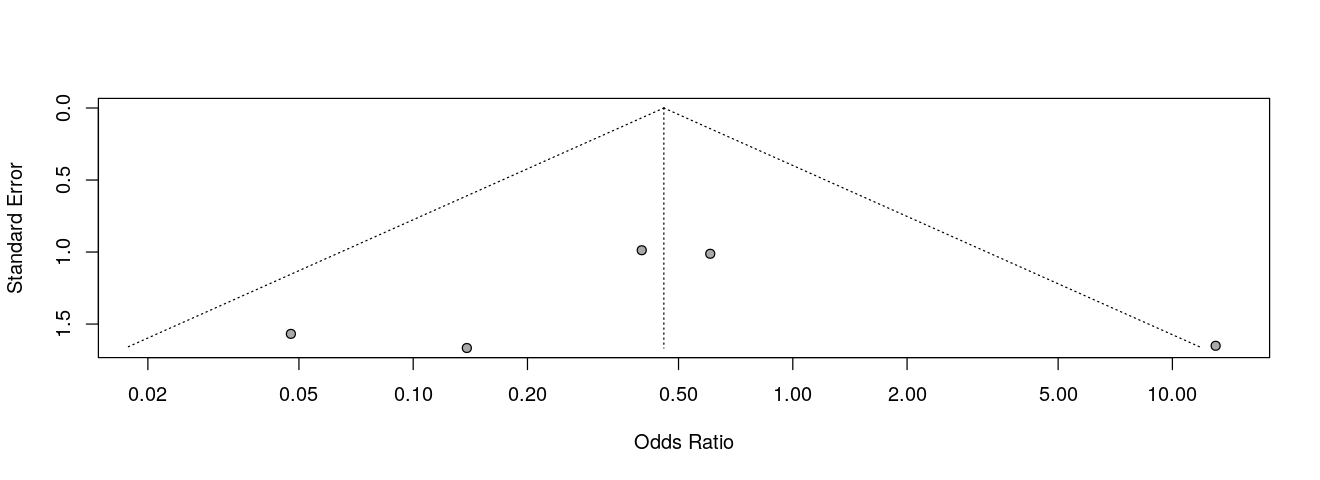


Figure S2. Funnel plot for complications endpoint

### **Subgroup analysis**

**Effect of the matrix type**


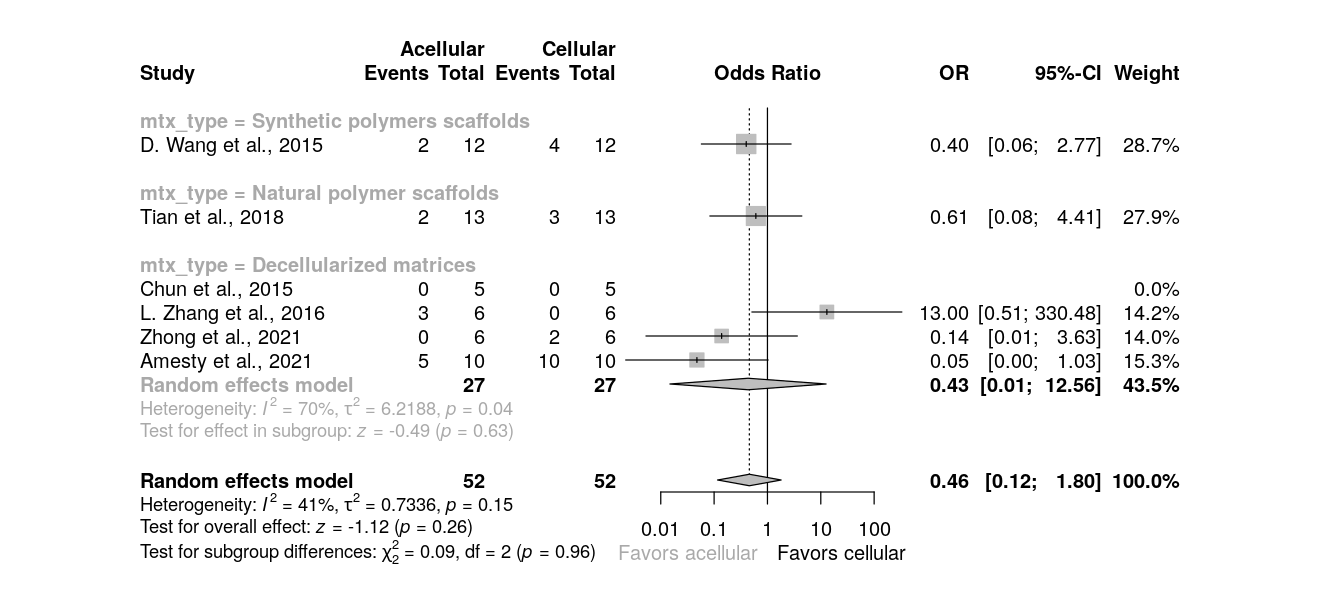


Figure S3. Subgroup analysis for complications endpoint: matrix type

### **Effect of the matrix material**

###
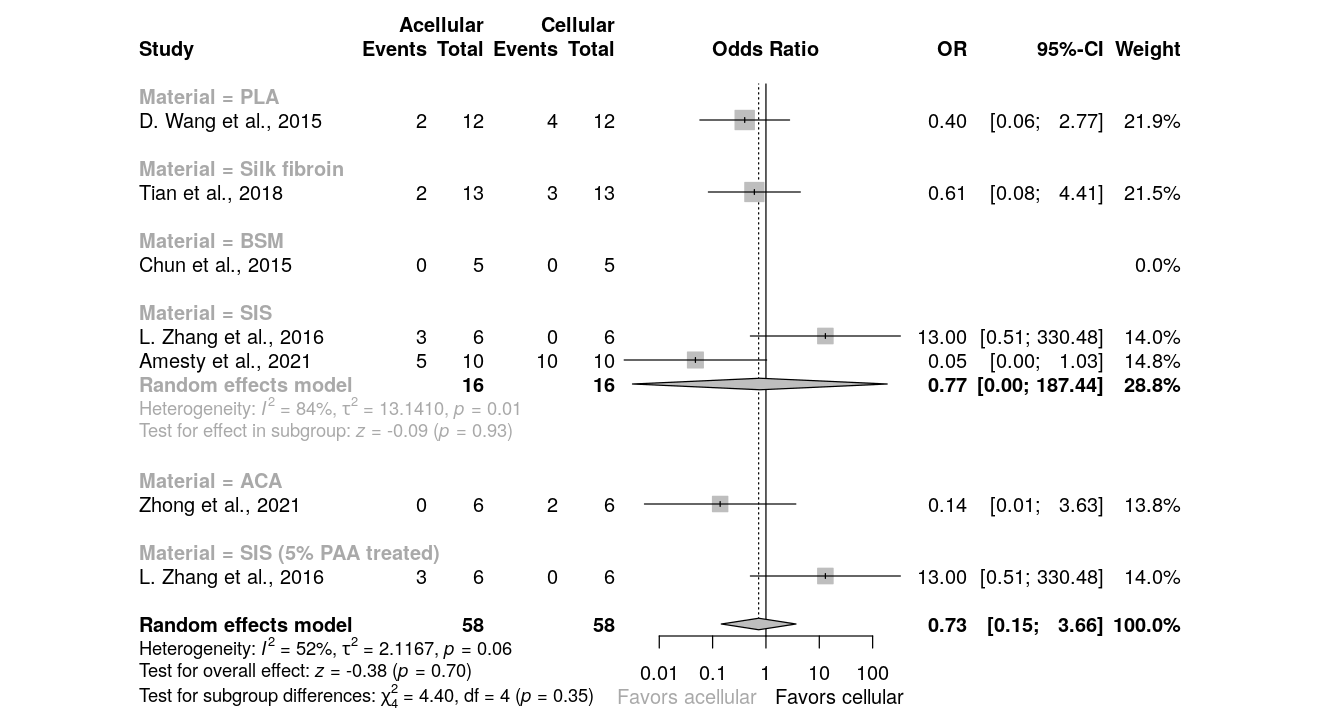


Figure S4. Subgroup analysis for complications endpoint: matrix material

### **Effect of Cell Type**

###
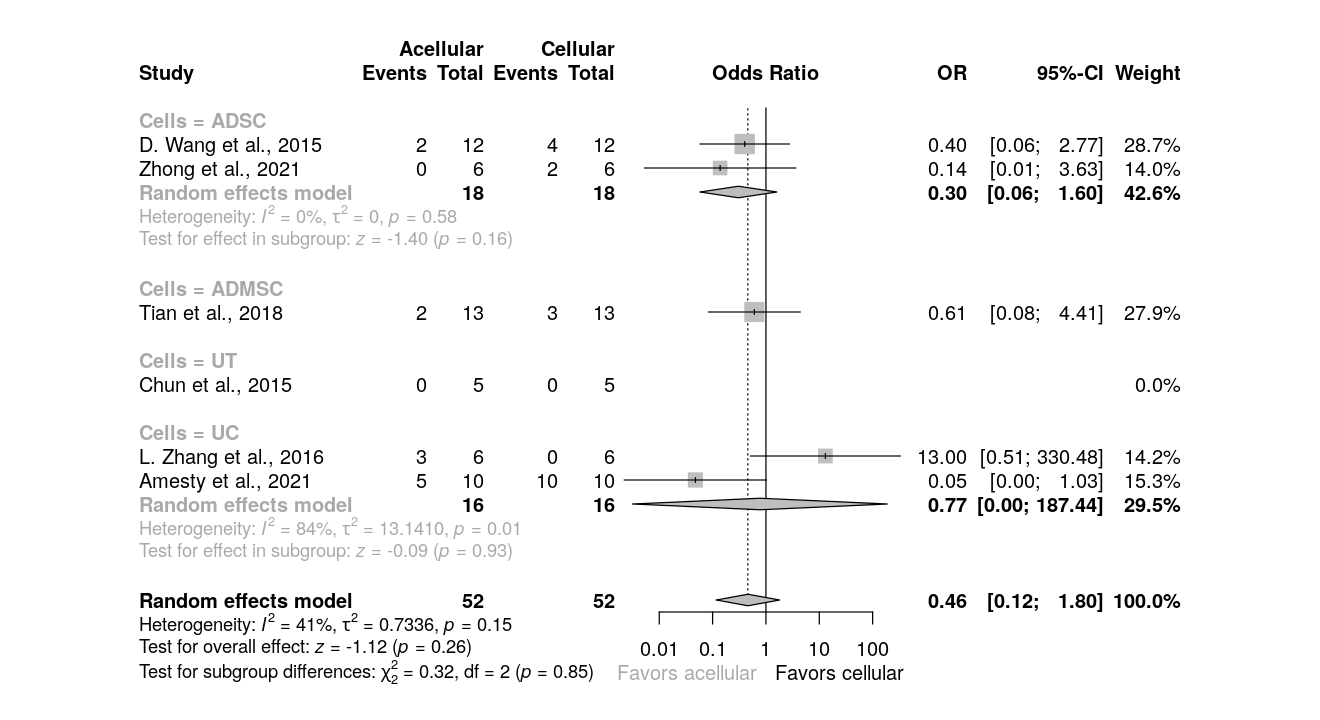


Figure S5. Subgroup analysis for complications endpoint: cell type

**Effect of the type of scaffold** **animal model**

###
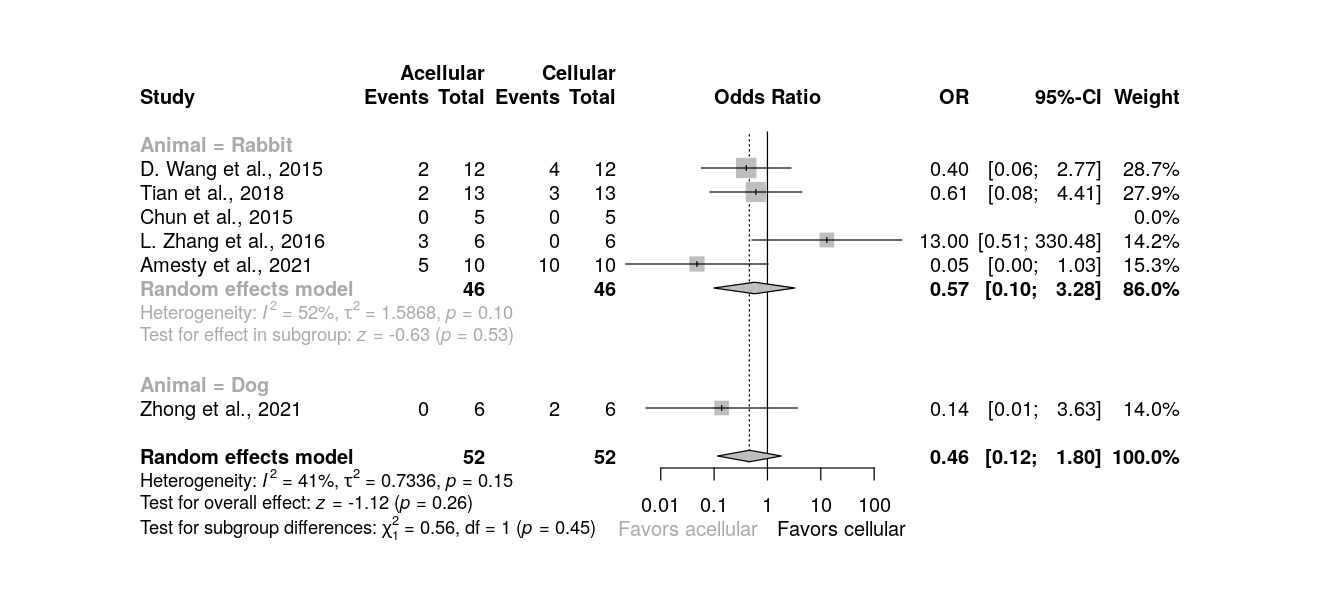


Figure S6. Subgroup analysis for complications endpoint: animal model

**Effect of the type of scaffold**

###
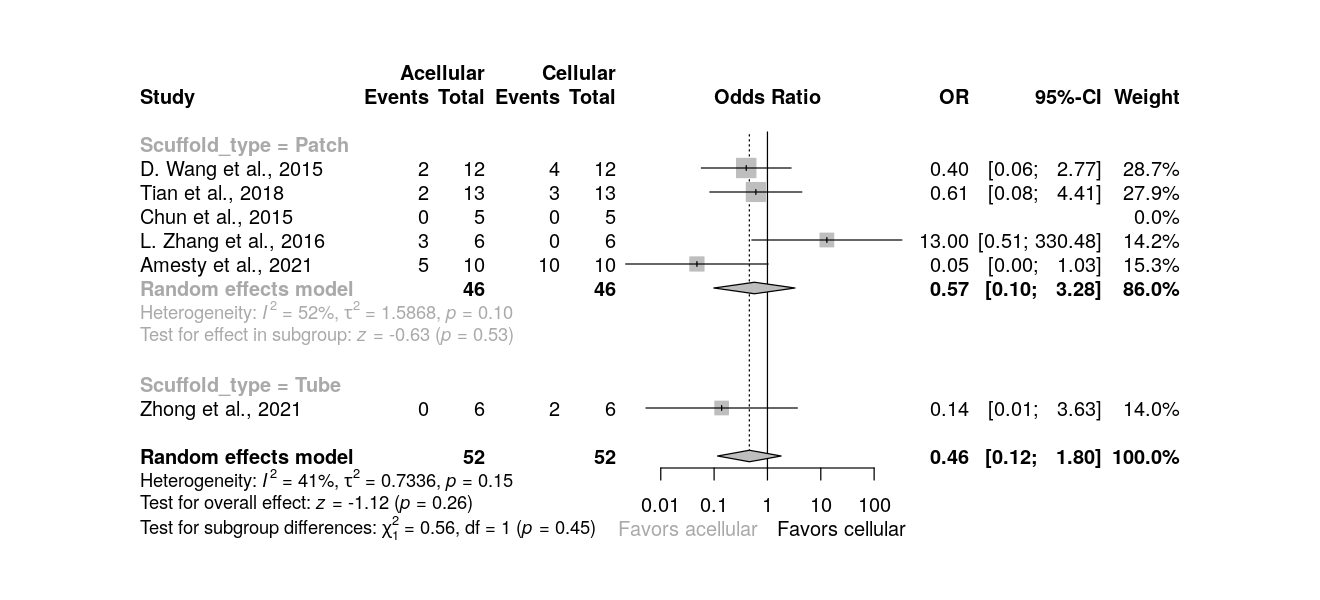


Figure S7. Subgroup analysis for complications endpoint: type of scaffold

**Length effect**

- meta-regression

Mixed-Effects Model (k = 5; tau^2 estimator: REML)

tau^2 (estimated amount of residual heterogeneity): 2.3865 (SE = 3.5491)

tau (square root of estimated tau^2 value): 1.5448

I^2 (residual heterogeneity / unaccounted variability): 55.23%

H^2 (unaccounted variability / sampling variability): 2.23

R^2 (amount of heterogeneity accounted for): 0.00%

Test for Residual Heterogeneity:

QE(df = 3) = 6.5993, p-val = 0.0858

Test of Moderators (coefficient 2):

QM(df = 1) = 0.2407, p-val = 0.6237

Model Results:

estimate se zval pval ci.lb ci.ub

intrcpt 0.4187 2.6156 0.1601 0.8728 -4.7078 5.5451

Length -0.0626 0.1276 -0.4907 0.6237 -0.3128 0.1875

---

Signif. codes: 0 ‘***’ 0.001 ‘**’ 0.01 ‘*’ 0.05 ‘.’ 0.1 ‘ ’ 1

## 2. Strictures

##
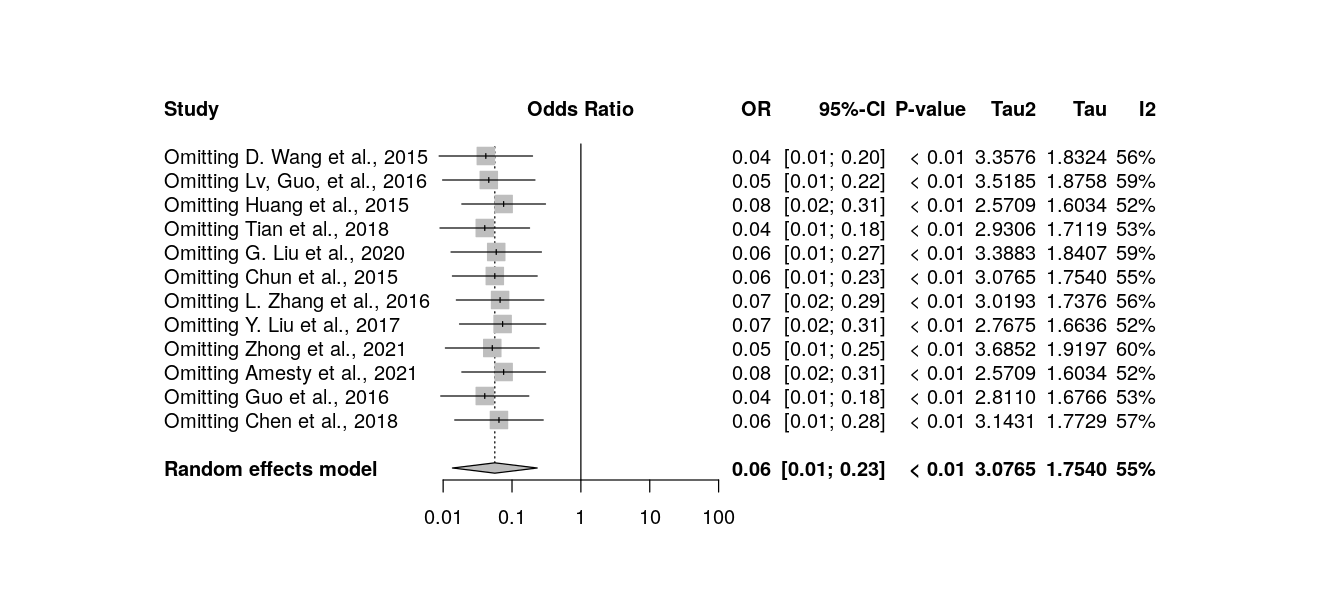


Figure S8. Sensitivity analysis (leave-one-out approach) for stricture endpoint

Peters’ test results

Test result: t = -0.31, df = 9, p-value = 0.7622

Sample estimates:

bias se.bias intercept se.intercept

-8.4318 27.0310 -3.1351 1.7913

Details:

- multiplicative residual heterogeneity variance (tau^2 = 22.5345)

- predictor: inverse of total sample size

- weight: inverse variance of average event probability

- reference: Peters et al. (2006), JAMA

##
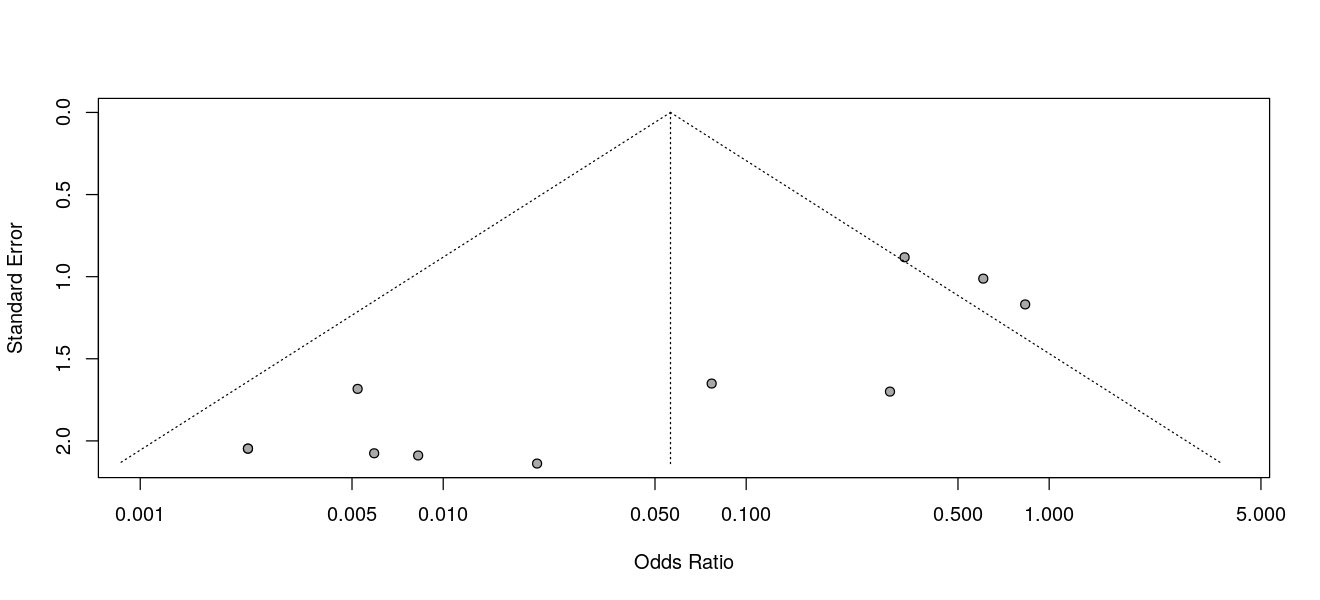


Figure S9. Funnel plot for stricture endpoint

## 2.1. Subgroup analysis

**Effect of the matrix type**


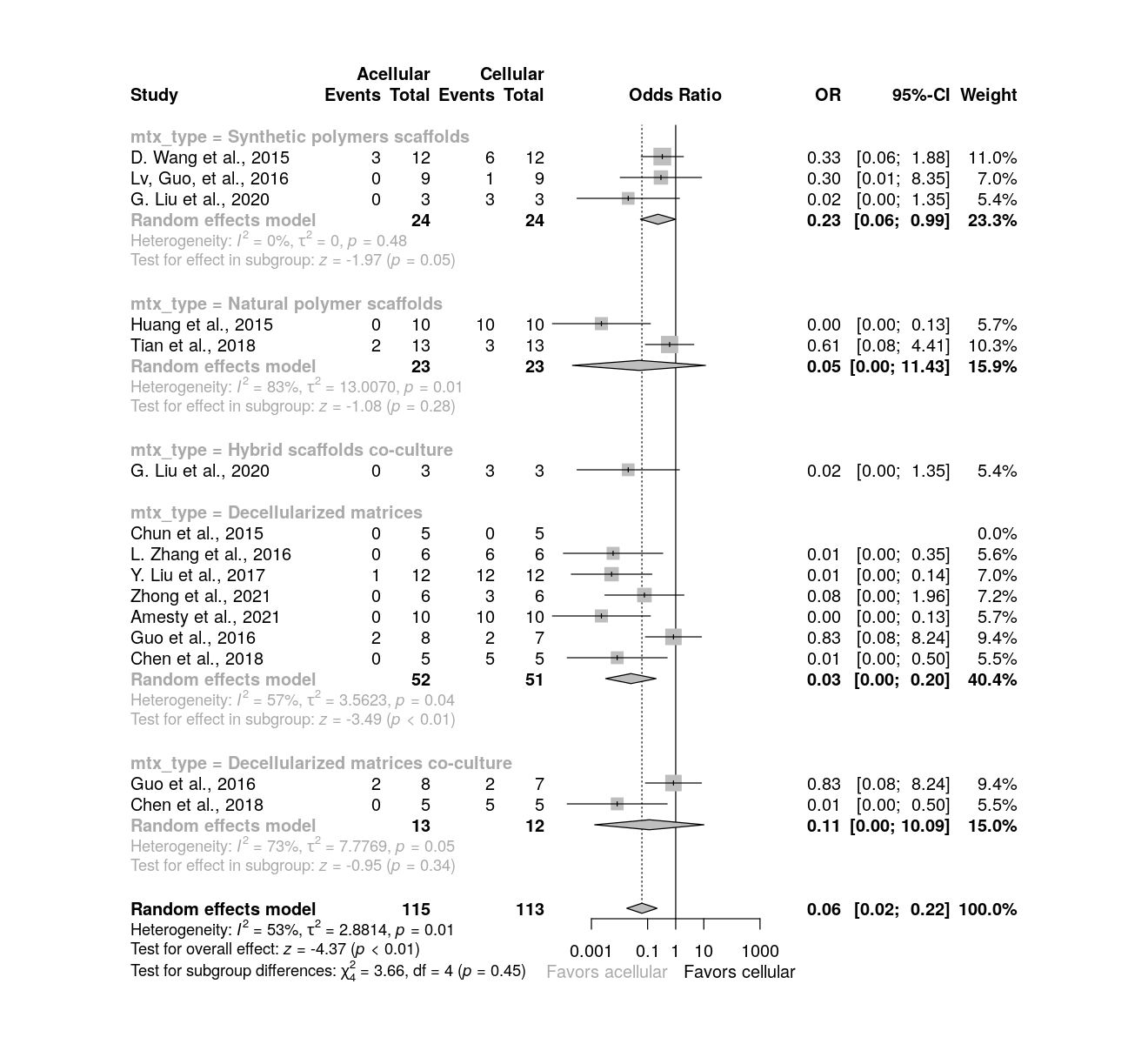


Figure S10. Subgroup analysis for stricture endpoint: matrix type

### **Effect of the matrix material**

###
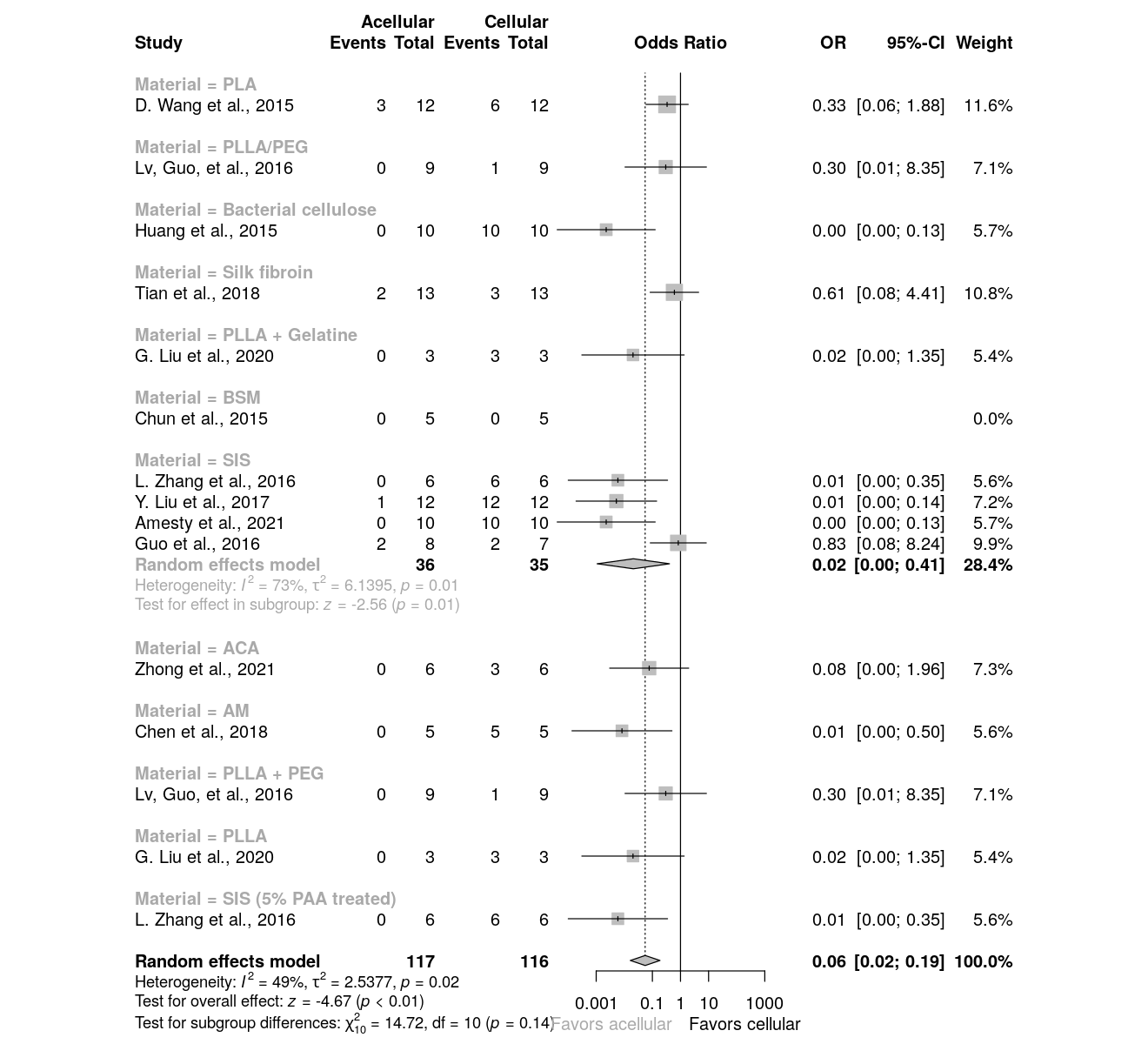


Figure S11. Subgroup analysis for stricture endpoint: matrix material

### **Effect of Cell Type**

###
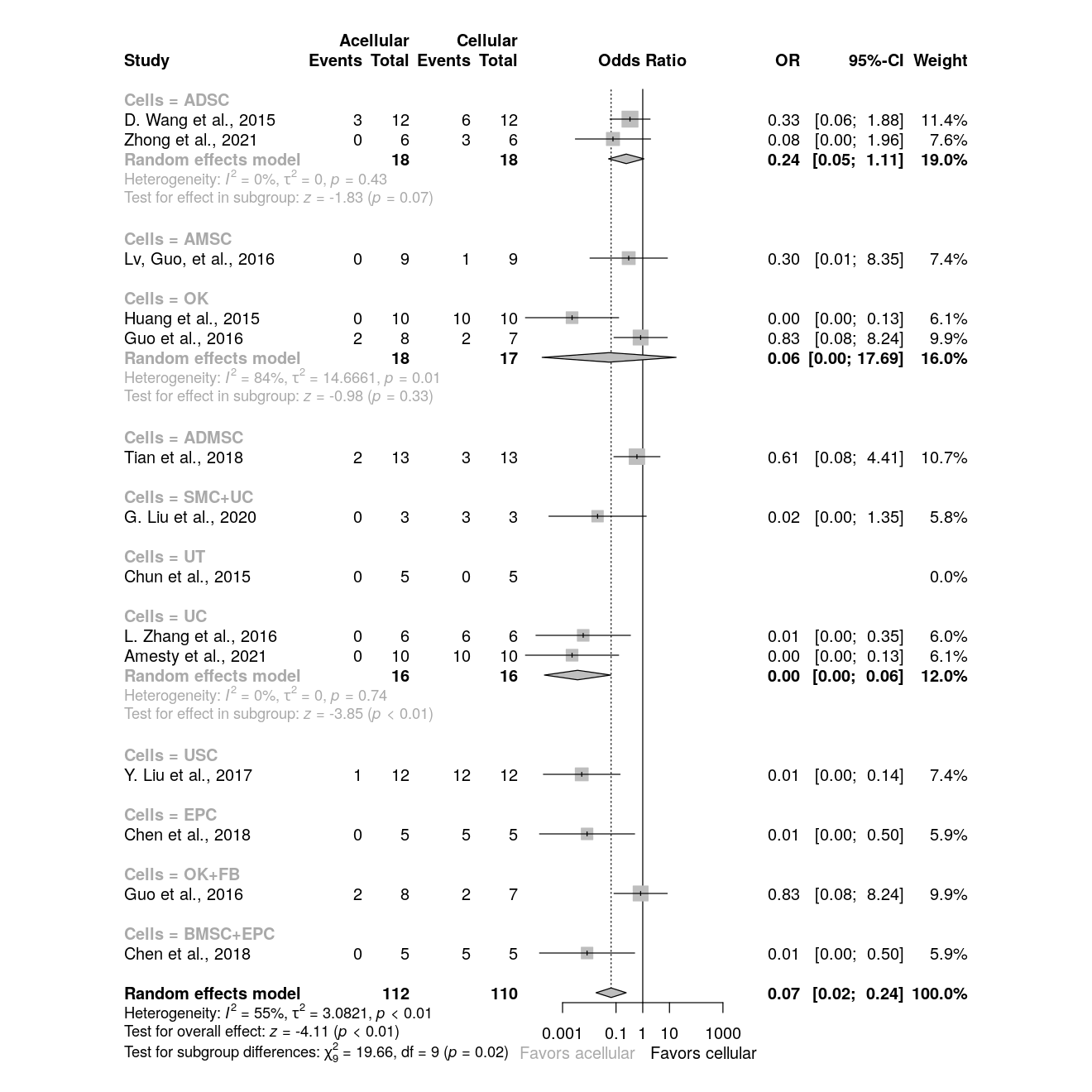


Figure S12. Subgroup analysis for stricture endpoint: cell type

**Effect of the type of scaffold** **animal model**

###
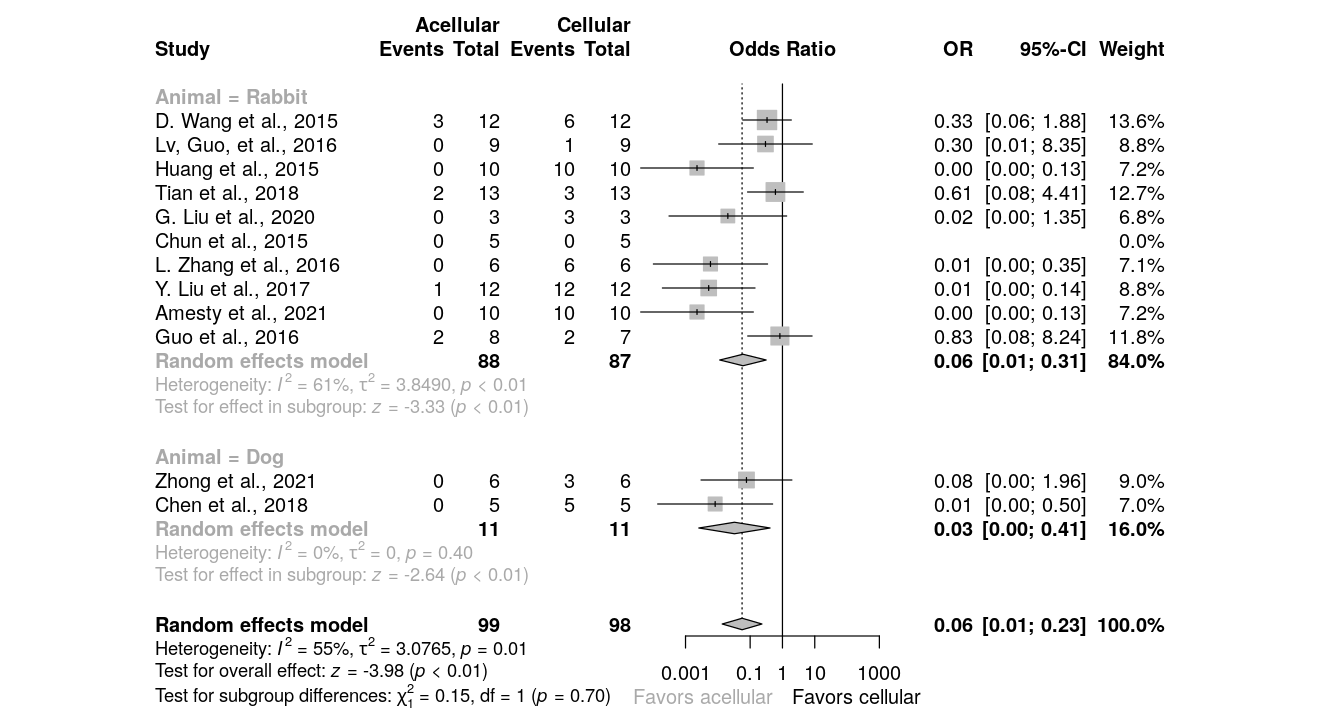


Figure S13. Subgroup analysis for stricture endpoint: animal model

**Effect of the type of scaffold**

###
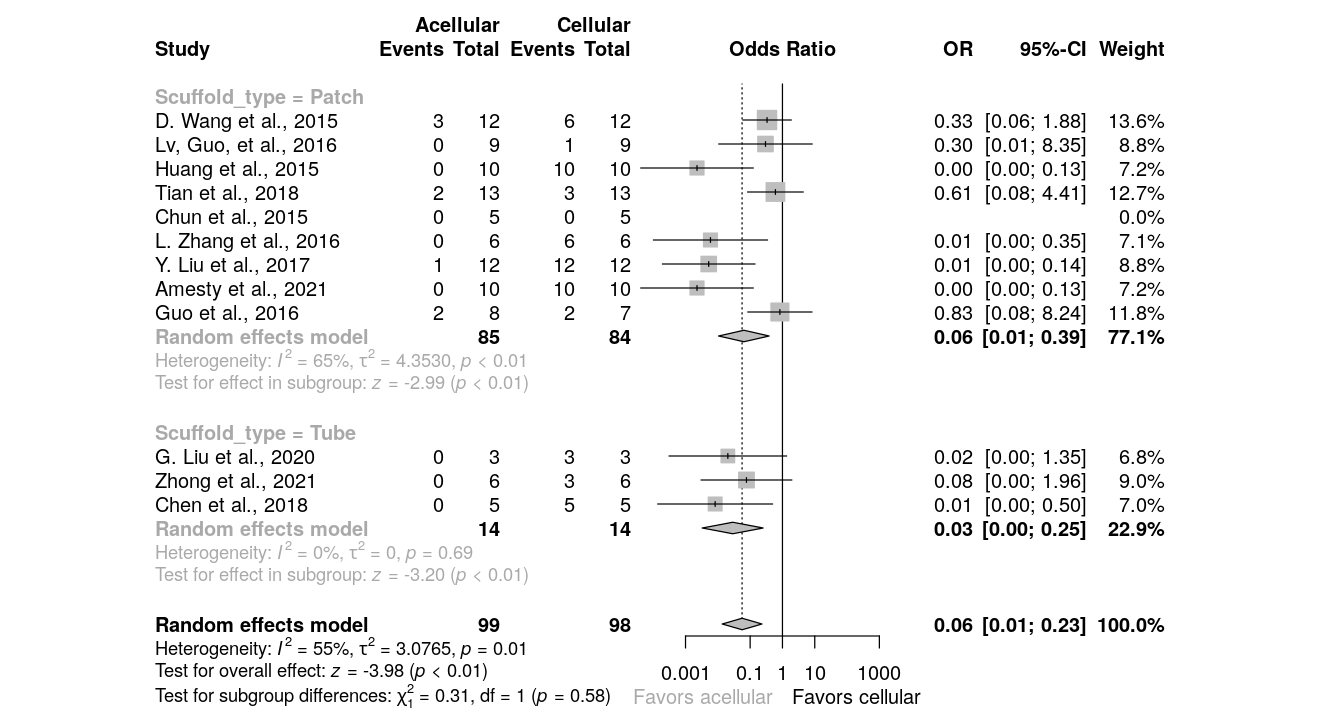


Figure S14. **Subgroup analysis for stricture endpoint: type of scaffold**

**Length effect**

- meta-regression

Mixed-Effects Model (k = 11; tau^2 estimator: REML)

tau^2 (estimated amount of residual heterogeneity): 3.7087 (SE = 3.0443)

tau (square root of estimated tau^2 value): 1.9258

I^2 (residual heterogeneity / unaccounted variability): 59.61%

H^2 (unaccounted variability / sampling variability): 2.48

R^2 (amount of heterogeneity accounted for): 0.00%

Test for Residual Heterogeneity:

QE(df = 9) = 22.0833, p-val = 0.0086

Test of Moderators (coefficient 2):

QM(df = 1) = 0.0296, p-val = 0.8634

Model Results:

estimate se zval pval ci.lb ci.ub

intrcpt -2.4675 2.7845 -0.8862 0.3755 -7.9249 2.9900

Length -0.0224 0.1301 -0.1720 0.8634 -0.2775 0.2327

---

Signif. codes: 0 ‘***’ 0.001 ‘**’ 0.01 ‘*’ 0.05 ‘.’ 0.1 ‘ ’ 1

## References

[^Borenstein]: Borenstein, Michael, Larry V Hedges, Julian PT Higgins, and Hannah R Rothstein. 2011. Introduction to Meta-Analysis. John Wiley & Sons.

[^peters]: Peters, Jaime L, Alex J Sutton, David R Jones, Keith R Abrams, and Lesley Rushton. 2006. “Comparison of Two Methods to Detect Publication Bias in Meta-Analysis.” JAMA 295 (6): 676–80

[^R]: R Core Team (2022). R: A language and environment for statistical computing. R Foundation for Statistical Computing, Vienna, Austria. URL <https://www.R-project.org/>.

[^Wan]: Wan X., Wang W., Liu J., and Tong T. (2014). Estimating the sample mean and standard deviation from the sample size, median, range and/or interquartile range. BMC Medical Research Methodology. 14:135.

[^detorri]: Dettori JR, Norvell DC, Chapman JR. Fixed-Effect vs Random-Effects Models for Meta-Analysis: 3 Points to Consider. Global Spine J. 2022 Sep;12(7):1624-1626. <https://doi.org/10.1177/21925682221110527>. Epub 2022 Jun 20. PMID: 35723546; PMCID: PMC9393987.

[^cochrane_handbook]: Higgins JPT, Thomas J, Chandler J, Cumpston M, Li T, Page MJ, Welch VA (editors). Cochrane Handbook for Systematic Reviews of Interventions version 6.3 (updated February 2022). Cochrane, 2022. Available from <https://www.training.cochrane.org/handbook>.

[^bonnet]: Bonett, D. G. (2008). Confidence intervals for standardized linear contrasts of means. Psychological Methods, 13(2), 99–109. <https://doi.org/10.1037/1082-989X.13.2.99>

[^begg]: Begg, C. B., & Mazumdar, M. (1994). Operating characteristics of a rank correlation test for publication bias. Biometrics, 50(4), 1088–1101. <https://doi.org/10.2307/2533446>

Supplementary Table 3. Clinical trials (2015 – 2022): Acellular tissue-engineered matrices for urethroplasty

| **№** | **Reference** | **Matrix** | **Number of**  **patients** | **Urethral stricture**  **length (cm)** | **Patch or tube** | **Mean**  **follow-up**  **(months)** | **Complications** | **Success rate %** |
| --- | --- | --- | --- | --- | --- | --- | --- | --- |
| 1 | Lin et al., 2020^1^ | ADM  Dartos | 35  80 | 3 | Patch | 9–15  (median: 11.56) | 13  43 | 89  93 |
| 2 | Mandal et al., 2020^2^ | Pericardial patch | Peno-bulbar -7  Penile – 2 | 5-18 | Patch | 2-12  (median: 8) | 0  0 | 86  100 |
| 3 | Razzaghi et al., 2020^3^ | AM | 28 | 9.6 +/-2.6 | Tube | 6–26  (median: 13.3 +/- 4.5) | 4 | 86 |
| 4 | Song et al., 2022^4^ | PLA | Bulbar - 16  Penile - 8  Bulbar + penile -1 | 1-7 | Patch | 33.56  (median: 16.50) | 0 | 88  63  100 |

**Аbbreviations:** **ADM** - acellular dermal matrix; **AM** - amniotic membrane; **PLA**  - polylactid acid.

Supplementary Table 4. Clinical trials (2015 – 2022): Cell seeded tissue-engineered matrices for urethroplasty

| **№** | **Reference** | **Matrix** | **Type of autologous cells** | **Number**  **of**  **patients** | **Urethral stricture length (cm)** | **Patch or Tube** | **Mean**  **follow-up**  **(months)** | **Complications** | **Success rate %** |
| --- | --- | --- | --- | --- | --- | --- | --- | --- | --- |
| 1 | Ram-Liebig et al., 2017^5^ | MukoCell^1^ | OK | 99 | 3,8 | Patch | 12  24 | 9 | 73  67 |
| 2 | Barbagli et al., 2018^6^ | MukoCell^1^ | OK | 38 | 1 - >7,1^2^ | Patch | 55 | 0 | 84 |
| 3 | Vaddi et al., 2019^7^ | TGF | OK | 6 | 2– 3,5 | Patch | 10 , 18, 36, 39, 40 | 2 | 67 |
| 4 | Karapanos et al., 2021^8^ | MukoCell^1^ | OK | 77 | 2-16 | Patch | 38 | 1 | 69 |

**Footnote:**

1 - Tissue-engineered oral mucosa graft (TEOMG) approved for sale in Germany (MukoCell®),

2 - 10 patients with strictures longer than 7.1 cm were included in the study.

**Аbbreviations**: **OK** - oral keratinocytes; **TGF** - thermoreversible gelation polymer scaffold.

1. Lin, D. *et al.* Use of Acellular Dermal Matrix for Urethroplasty Coverage in Proximal Hypospadias Repair: a Pilot Study. *Adv. Ther.* **37**, 1425–1435 (2020).

2. Mandal, T. K. *et al.* Tissue engineered indigenous pericardial patch urethroplasty: A promising solution to a nagging problem. *Asian J. Urol.* **7**, 56–60 (2020).

3. Razzaghi, M. *et al.* Use of human amniotic membrane repair of anterior urethral defect: First clinical report. *Int. J. Urol.* **27**, 605–609 (2020).

4. Song, L. *et al.* Anterior substitutional urethroplasty using a biomimetic poly-l-lactide nanofiber membrane: Preclinical and clinical outcomes. *Bioeng. Transl. Med.* (2022) doi:10.1002/btm2.10308.

5. Ram-Liebig, G. *et al.* Results of Use of Tissue-Engineered Autologous Oral Mucosa Graft for Urethral Reconstruction: A Multicenter, Prospective, Observational Trial. *EBioMedicine* **23**, 185–192 (2017).

6. Barbagli, G. *et al.* Anterior Urethroplasty Using a New Tissue Engineered Oral Mucosa Graft: Surgical Techniques and Outcomes. *J. Urol.* **200**, 448–456 (2018).

7. Vaddi, S. P., Reddy, V. B. & Abraham, S. J. Buccal epithelium Expanded and Encapsulated in Scaffold-Hybrid Approach to Urethral Stricture (BEES-HAUS) procedure: A novel cell therapy-based pilot study. *Int. J. Urol.* **26**, 253–257 (2019).

8. Karapanos, L. *et al.* Safety and mid-term surgical results of anterior urethroplasty with the tissue-engineered oral mucosa graft MukoCell®: A single-center experience. *Int. J. Urol.* (2021).
